# Citrullination of histone H3 drives IL-6 production by bone marrow mesenchymal stem cells in MGUS and multiple myeloma

**DOI:** 10.1038/leu.2016.187

**Published:** 2016-08-12

**Authors:** G McNee, K L Eales, W Wei, D S Williams, A Barkhuizen, D B Bartlett, S Essex, S Anandram, A Filer, P A H Moss, G Pratt, S Basu, C C Davies, D A Tennant

**Affiliations:** 1 Institute of Metabolism and Systems Research, College of Medical and Dental Sciences, University of Birmingham, Birmingham, UK; 2 Institute of Cancer and Genomic Sciences, College of Medical and Dental Sciences, University of Birmingham, Birmingham, UK; 3 Department of Haematology, Royal Wolverhampton NHS Trust, Wolverhampton, UK; 4 Institute of Immunology and Immunotherapy, College of Medical and Dental Sciences, University of Birmingham, Birmingham, UK; 5 Department of Haematology, University Hospitals Birmingham NHS Trust, Birmingham, UK

## Abstract

Multiple myeloma (MM), an incurable plasma cell malignancy, requires localisation within the bone marrow. This microenvironment facilitates crucial interactions between the cancer cells and stromal cell types that permit the tumour to survive and proliferate. There is increasing evidence that the bone marrow mesenchymal stem cell (BMMSC) is stably altered in patients with MM—a phenotype also postulated to exist in patients with monoclonal gammopathy of undetermined significance (MGUS) a benign condition that precedes MM. In this study, we describe a mechanism by which increased expression of peptidyl arginine deiminase 2 (PADI2) by BMMSCs in patients with MGUS and MM directly alters malignant plasma cell phenotype. We identify PADI2 as one of the most highly upregulated transcripts in BMMSCs from both MGUS and MM patients, and that through its enzymatic deimination of histone H3 arginine 26, PADI2 activity directly induces the upregulation of interleukin-6 expression. This leads to the acquisition of resistance to the chemotherapeutic agent, bortezomib, by malignant plasma cells. We therefore describe a novel mechanism by which BMMSC dysfunction in patients with MGUS and MM directly leads to pro-malignancy signalling through the citrullination of histone H3R26.

## Introduction

Multiple myeloma (MM) is a malignant disease of plasma cells within the bone marrow that, despite improving survival, remains incurable meaning an urgent requirement for novel therapies. One characteristic feature of MM is that a pre-malignant condition known as monoclonal gammopathy of undetermined significance (MGUS) can be present for many years prior to development of overt disease.^1^ No specific genetic alterations have yet been identified that distinguish patients with MGUS from those with MM, suggesting that another factor, such as transformation of the microenvironment are likely drivers of progression.^2, 3^ Malignant plasma cells have an absolute requirement for localisation within the bone marrow niche, which leads to a characteristic feature of MM: the relative rarity of disease metastasis to sites outside the marrow microenvironment. This suggests that, in common with most malignancies, stromal support of cancer cell survival in MM is important for maintaining and even driving disease.^4, 5, 6^

Key cellular components of both the normal and transformed bone marrow niche are the bone marrow mesenchymal stem cells (BMMSCs). There is now significant evidence to suggest that BMMSCs from patients with MM^7, 8, 9, 10, 11^ and MGUS^7^ are phenotypically different from those derived from healthy patients. Indeed they have not only been shown to exhibit a stably altered transcriptional profile,^7^ but may also contain a number of genetic lesions.^12^ The phenotypic changes resulting from this altered transcriptome appear to permit increased stromal support of the malignant plasma cell, and decreased osteoblastic differentiated function (bone mineralisation).^7, 8, 9, 10, 11^ The former is through both physical (that is, cell–cell) interactions, and secretion of a number of paracrine factors that support malignant plasma cell survival, proliferation, migration and chemoresistance.^5, 6, 13^ Although a number of signalling pathways have been implicated in the differentiation of BMMSCs there remains a lack of clarity as to their role.

A number of cytokines and chemokines have been shown to be involved in the cross-talk between the bone marrow stroma and malignant plasma cells, including interleukin-6 (IL-6),^14^ C-X-C motif chemokine 12 (CXCL12, also known as stromal-derived factor 1),^15^ vascular endothelial growth factor (VEGF),^16^ fibroblast growth factor,^17^ tumour necrosis factor α,^18^ interleukin-1β^18^ and cMET (also known as hepatocyte growth factor/scatter factor).^19^ IL-6 secretion by the cells of bone marrow microenvironment, especially the BMMSC population,^7, 9, 11^ has been shown to have a role in malignant plasma cell phenotype through increasing resistance to cell death stimuli and driving proliferation,^20^ as well as downregulating differentiation markers, such as CD38 and CD138.^21^ It is therefore now clear that through both physical association and paracrine interactions, signalling between BMMSCs and myeloma cells underlies the requirement of myeloma cells for BM localisation.

One defining feature of BMMSCs isolated from patient bone marrows is that they retain a transcriptomic profile despite being in culture.^7, 8, 9, 11, 21^ This suggests that they sustain this transcriptome through a stable genetic and/or epigenetic profile. Interestingly, little is known about the epigenetic status of BMMSCs in healthy individuals or of those from MGUS or MM patients. A post-translational modification in which there is increasing interest in both cancer and inflammation is the citrullination of peptidyl arginine, which can occur on many different protein species, including histones.^22^ These reactions are performed by the calcium-dependent peptidyl arginine deiminase (PADI) family of enzymes of which there are five members (PADI1, 2, 3, 4 and 6) with tissue-specific expression patterns.^23, 24, 25^ Peptidyl arginine deiminase 2 (PADI2) is ubiquitously expressed, and in many tissues has been reported as the major family member providing peptidyl arginine deiminase activity.^26^ Importantly, both PADI2 and PADI4 have been shown to be able to alter gene transcription through the citrullination of residues in the histone tails:^27, 28, 29^ a modification that may be physiologically irreversible owing to the lack of any known peptidylcitrulline imidating enzyme. This modification is therefore a good candidate for the creation of a long-term change in the cellular transcriptome, as noted in the BMMSCs of MM patients.

As MM is effectively incurable, and is now accepted to almost always arise from MGUS,^1^ the phenotype of cells contributing to this benign condition may provide important clues as to factors determining progression to MM. We show here that BMMSCs from patients with both MGUS and MM exhibit a significant shift in their transcriptome compared with those from healthy individuals. In addition, we demonstrate that one of the most upregulated transcripts in BMMSCs from MGUS and MM, PADI2 is responsible for mediating increased IL-6 expression from these cells through the deimination of arginine residue 26 of histone H3 (H3R26) to form citrulline (H3R26cit). This increased IL-6 production is sufficient to drive resistance of malignant plasma cells to bortezomib, a highly clinically relevant anti-myeloma drug. We therefore demonstrate a novel mechanism for BM microenvironment-induced acquisition of drug resistance in MM and importantly, that this phenotype is already present in patients with the apparently benign MGUS.

## Materials and methods

### Patient sample collection, BMMSC isolation and cell culture

Patients with MM and MGUS were recruited from specialist clinics at The Royal Wolverhampton Hospitals NHS Trust and Heart of England NHS Trust, UK. The study received approval from the local ethics committees at South Birmingham, Birmingham East, North, and Solihull, and informed written consent was obtained in accordance with the Declaration of Helsinki in all cases. For patients with MGUS/MM, BM aspirates from the iliac crest were diluted in MACS buffer for removal of the CD138^+^ cells using anti-CD138^+^ antibody-linked magnetic beads (Miltenyi Biotech, Bisley, UK), before the remaining cellular fraction was diluted in complete medium, and BMMSCs allowed to adhere to tissue culture plates. Control BMMSCs for the transcriptomic study were obtained by flushing out a section of rib from patients undergoing thoracotomies with Rosewell Park Memorial Institute (RPMI) 1640 medium (Sigma, Gillingham, UK). Around 80% of the patients recruited to this control group had a primary lung tumour: none had bone marrow involvement or had received chemotherapy. Patient number and characteristics for the transcriptomic study are shown in Supplementary Table 1, whereas those of the BMMSCs used in all other studies are shown in Supplementary Table 2. For the other studies, ‘healthy' BMMSCs were isolated from patients undergoing hip surgery, with informed written consent obtained and samples collected through the University of Birmingham Research Tissue Bank (HTA license 12358, REC reference 09/H1010/75). Bone marrow aspirates from the femoral head were diluted in RPMI1640 and the BMMSCs in the non-fatty layer allowed to adhere to tissue culture plates. BMMSCs and the HS5 human bone marrow fibroblast cell line (ATCC CRL11882, LGC Standards, Teddington, UK, purchased December 2013, tested for mycoplasma every 3 months by PCR) were cultured in RPMI1640 medium with 10% fetal bovine serum (Hyclone, GE Healthcare, Little Chalfont, UK) at 37 °C in a humidified incubator with 5% CO_2_. BMMSCs were allowed to adhere and proliferate to ~80% confluence before harvesting. Wild-type or active-site mutant PADI2 (C647S), each with N-terminal FLAG tag, were expressed through transient transfection of the complementary DNAs within the pcDNA3 vector (or empty vector control) using jetPEI (Polyplus transfection, Illkirch, France) for 24 h before analysis. Where shown, the pan-PADI inhibitor Cl-Amidine (200 μm, Caymen Chemical, Ann Arbor, MI, USA) was used for 48 or 72 h. Small interfering (siRNA) against PADI2 at 20 nm (Oligo 1: AGCCTTGACTCATTTGGAA, and Oligo 4: AAGCGAATCACCATCAACA) or PADI4 (GCGAAGACCTGCAGGACAT) were transfected using TransIT-TKO (Mirus Bio, Madison, WI, USA) and assayed at the time point shown.

### Transcriptomic and Real-time PCR analyses

At passage 3, mRNA from BMMSCs (Supplementary Table 1) was extracted using the RNeasy mini kit (Qiagen, Düsseldorf, Germany). mRNA was prepared and hybridised to Affymetrix HuEx-1_0-st-v2 exon arrays (Affymetrix, Santa Clara, CA, USA) as per manufacturer's instructions. Gene level analysis of the Affymetrix exon array was performed using Affymetrix Expression Console with the default settings of ‘Extended: RMA-Sketch' and Affymetrix annotation na32. Differentially expressed genes were identified using the limma package^30^ with cutoff values of *P*<0.001 and absolute fold change>1.5. Heatmaps were generated using dChip (http://www.dchip.org/) with the default settings. Full data set is available in the Gene Expression Omnibus repository with accession number GSE80608. For Taqman analyses, RNA was isolated using the RNeasy kit before reverse transcription (AMV reverse transcription kit, Promega, Southampton, UK). Taqman real-time PCR was performed on an ABI7500; using primer/probe mixes (Supplementary Table 3, Life Technologies, Inchinnan, UK). Cycle threshold was automatically determined by ABI7500 software and expression levels were normalised to ACTB and calculated relative to control. All transcript data are presented as median-centred box and whisker (10–90th percentile) plots.

### Detection of apoptosis by flow cytometry

Primary BMMSCs from MGUS patients that showed high or low PADI2 protein levels (Supplementary Table 2) were seeded at 5 × 10^5^ cells 24 h before 10^6^ JJN3 cells were added to a TransWell insert (Costar Appleton Woods, Birmingham, UK), 24 h prior to treatment with 10 nm bortezomib. After a further 24 h, JJN3 cell apoptosis was assessed by Annexin V/propidium iodide staining as per manufacturer's instructions (Life Technologies) and flow cytometry (LSRII; BD Biosciences, Oxford, UK). The resulting data were gated and quantified (Flow Jo v.7.6.5) and presented after subtraction of background apoptosis of untreated, monocultured JJN3 cells. Where shown, BMMSCs were treated with Cl-Amidine (200 μm, 16 h), which was removed before washing and subsequent incubation with JJN3 cells (within the TransWell insert). Experiments were performed three times and expression analysed in triplicate; data presented as mean±s.e.m.

### Measurement of IL-6

HS5 cell culture supernatants were harvested after treatment with either Cl-Amidine or siRNA and centrifuged to remove dead cells. Supernatants were analysed using an IL-6 detection kit as per manufacturer's instructions (eBioscience, Altrincham, UK). Experiments were performed three times in triplicate; data are presented as mean±s.d. IL-6 from BM plasma was detected using a multiplex bead analysis approach (Affymetrix). Measurements were performed as per manufacturer's instructions. In brief, plasma samples, diluted with an equal volume of phosphate-buffered saline, pH7.4, were incubated with monoclonal antibody-coated capture beads for 1 h at 20 °C. Washed beads were further incubated with biotin-labelled polyclonal anti-human cytokine antibody for 30 min, streptavidin–phycoerythrin for 30 min and analysed on a Luminex 100 bioanalyser (Luminex, Ballina, Ireland) and converted to absolute concentrations using standard curves. Data are presented as median-centred box and whisker (10–90th percentile) plots.

### Chromatin Immunoprecipitation

Chromatin immunoprecipitation was performed as previously described.^31^ In brief, HS5 fibroblasts were cross-linked with 1% formaldehyde before neutralisation with 0.125 m glycine. After lysis (10 mm Tris-HCl, pH8.0, 10 mm NaCl, 0.2% NP-40, 10 mm sodium butyrate, 50 μg/ml phenylmethylsulfonyl fluoride and 1 μg/ml leupeptin), nuclei were purified, lysed (50 mm Tris-HCl, pH8.1, 10 mm ethylenediaminetetraacetic acid, 1% sodium dodecyl sulphate, 10 mm sodium butyrate, 50 μg/ml phenylmethylsulfonyl fluoride and 1 μg/ml leupeptin) and diluted in immunoprecipitation buffer (20 mm Tris-HCl, 150 mm NaCl, 2 mm ethylenediaminetetraacetic acid, 1% Triton X-100, 0.01% sodium dodecyl sulphate, 10 mm sodium butyrate, 50 μg/ml phenylmethylsulfonyl fluoride and 1 μg/ml leupeptin). DNA was sheared to 300–1000 bp, and diluted further in immunoprecipitation buffer. After pre-clearing (normal rabbit immunoglobulin G), chromatin was co-immunoprecipitated with anti-histone H3 trimethyl K4 (Abcam, Cambridge, UK, ab8580) or anti-histone H3 cit26 (Abcam; ab19847, lot GR146412-1). After washing and elution of protein-DNA complexes from the beads (100 mm NaHCO_3_, 1% sodium dodecyl sulphate), cross-links were reversed by heating, and treated with proteinase K. DNA was purified; primers used to quantify DNA are shown in Supplementary Table 4. Experiments were performed three times and PCR performed in triplicate; data presented as mean±s.e.m.

### Western blotting

Samples were harvested, washed in phosphate-buffered saline and after pelleting, lysed directly in lysis buffer (10 mm HEPES/KOH pH7.4, 10 mm KCl, 0.05% NP-40, 1 mm sodium orthovanadate). After centrifugation of the lysate, the supernatant containing the cytoplasmic components was retained, and the pelleted nuclei were washed with lysis buffer before extraction of the nucleoplasmic protein fraction in low salt lysis buffer (10 mM TRIS/HCl pH7.4, 0.2 mm MgCl_2_). After blocking, membranes were probed with antibodies against actin (AC-40; Sigma), R26cit (ab19847; Abcam), R2,8,17cit (ab5103; Abcam), PADI2 (ab16478; Abcam) or PADI4 (ab128086; Abcam), followed by the appropriate horseradish peroxidase-linked secondary antibody (Cell Signalling, NEB, Hitchin, UK) and visualised using ECL Prime (GE Healthcare, Amersham, UK).

### Statistics

Sample size for patient studies could not be pre-determined because of the lack of similar data on healthy and MGUS populations. No samples were excluded from analyses presented. Error bars are shown for all data presented. All statistics were performed using GraphPad Prism v.6.07. In order to compare different patient groups without assumptions of data distribution, non-parametric tests were used (Mann–Whitney and Kruskal–Wallis) with Dunn's *post test* comparisons where appropriate. Where statistical tests were used on data from cell culture experiments, student's two-tailed *t*-tests with Welch's correction were used to avoid assumptions of variance between groups.

## Results

**Figure 1:**
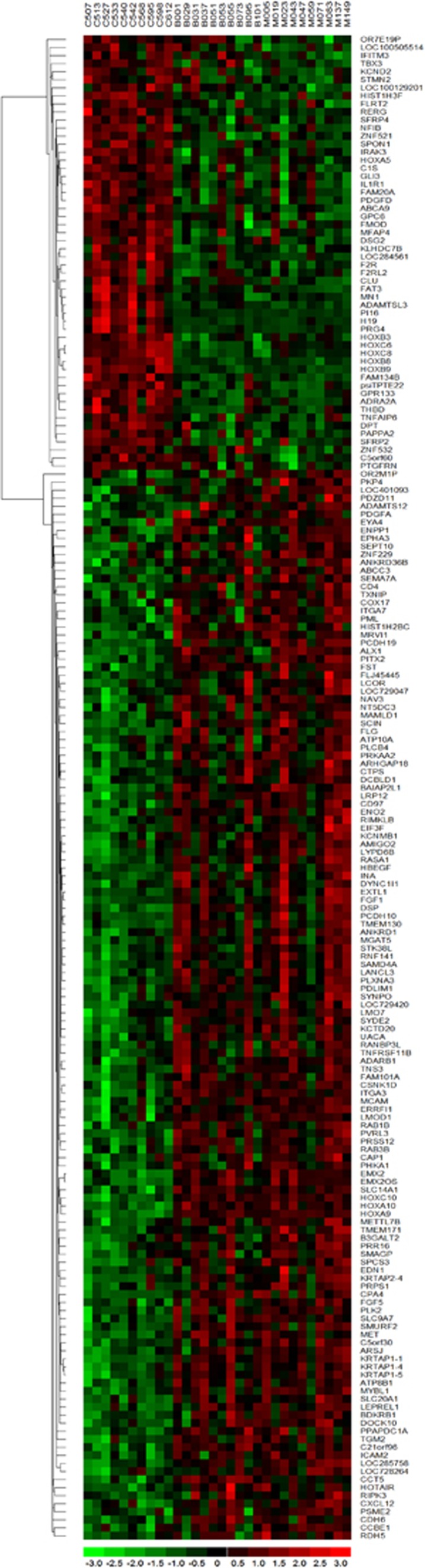
Heatmap of transcriptomic analysis from 30 BMMSC samples, 10 each from control, MGUS and MM patients shows that cells from patients with MGUS and MM cluster together, and are different from those isolated from healthy individuals. One hundred and eighty seven genes were identified with a cutoff for gene expression change of greater than 1.5-fold, and *P*<0.001.

**Figure 2:**
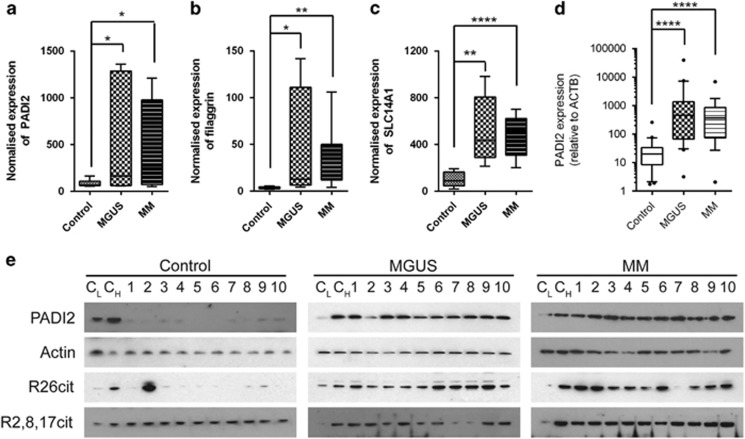
(**a**) Three of the top five upregulated transcripts that differed between control and ‘disease' (MGUS/MM) BMMSCs identified from the transcriptomic analysis were the peptidyl arginine deiminase, PADI2 (**a**), filaggrin (**b**) and the urea transporter, SLC14A1 (**c**). (**d**) PADI2 mRNA expression in BMMSCs from controls, MGUS and MM patients compared with beta-actin, analysed using Taqman, plotted on a logarithmic scale. Significant changes shown were calculated using Mann–Whitney tests. *P<0.05, **P<0.005, ****P<0.0001. (**e**) BMMSCs derived from patients with MGUS and MM have greatly increased expression of PADI2 and citrullination of histone H3 on residue R26 (R26cit) compared with healthy controls. Citrullination of other arginine residues on histone H3 (R2,8,17) under the control of PADI4 are not similarly consistently increased. ‘C_L_' and ‘C_H_' indicate control samples (C_L_: low expressing, C_H_: high expressing) analysed on each blot in order for blots to be more easily compared.

There is strong evidence to suggest that BMMSCs from patients with MM show a transformed phenotype: supporting malignant plasma cell survival, proliferation, and evasion of chemotherapy-mediated cell death.^7, 8, 9, 10, 11^ The BMMSC phenotype in MGUS patients is less-well characterised.^7^ We therefore performed a transcriptomic analysis of mRNA extracted from BMMSCs cultured from controls, MGUS and MM patients. BMMSCs isolated from patients with both MGUS and MM show markedly different gene expression profiles compared with the controls (Figure 1, cutoffs used, *P*<0.001, fold change 1.5), consistent with the previous report.^7^ Although a large number of transcripts were found to be differentially expressed between controls and MGUS (91) or MM (117; cutoff *P*<0.001, fold change of >2.0; Supplementary Tables 5 and 6), none were found to vary between MGUS and MM using the same statistical cutoffs, and only 32 if these were relaxed (cutoff of *P*<0.01, fold change of >1.3; Supplementary Table 7). Pathway analysis of differentially expressed genes between disease and control groups demonstrated that the most prominent enriched signalling pathway was WNT (*P*=0.00012 and 0.00002 for MM vs control and MGUS vs control, respectively; Supplementary Tables 8 and 9 and Supplementary Figure 1a), which has been previously implicated in the pathogenesis of MM.^32, 33, 34^ In addition, within the top five upregulated transcripts in MGUS and MM was a member of the PADI family of enzymes, PADI2 (Figure 2a), which was upregulated over fivefold in both MGUS and MM patient BMMSCs. Importantly, two other functionally related transcripts were also in the top five; filaggrin, a well-described target of PADI2 (Figure 2b), and SLC14A1, which encodes the urea transporter required for excretion of ammonium produced through PADI2 activity (Figure 2c, Supplementary Figure 1b). Interestingly, we recently published evidence that urea concentrations are significantly increased in the bone marrow plasma of patients with both MGUS and MM, consistent with significantly increased cellular production of ammonium, perhaps through PADI2 activity, in the bone marrow niche.^35^

To confirm the increased expression of PADI2 in BMMSCs in MGUS and MM, we assessed both mRNA and protein expression in BMMSCs isolated from an independent cohort of healthy individuals, MGUS and MM patients (Supplementary Table 2). We found that mRNA expression of PADI2 was relatively low in BMMSCs from healthy patients (Figure 2d), corresponding with an almost undetectable protein expression (Figure 2e). However, in BMMSCs from MGUS or MM patients, expression of PADI was greatly increased (Figures 2d and e): indeed mRNA expression was increased >30-fold. Corresponding mRNA expression of other PADI family members was unchanged (Supplementary Figure 2). We therefore assessed the activity of PADI2 through its citrullination of a recently described target: histone H3R26cit.^27^ We found that this modification of histone H3 was significantly increased in BMMSCs from MGUS and MM compared to healthy individuals (Figure 2e), where it was almost universally absent. In contrast, citrullination of other arginine residues in histone H3 that represent more specific PADI4 targets (R2, R8 and R17) were not upregulated to the same extent, suggesting that PADI2 activity, rather than PADI4 was likely to be responsible for H3R26cit levels observed.

**Figure 3:**
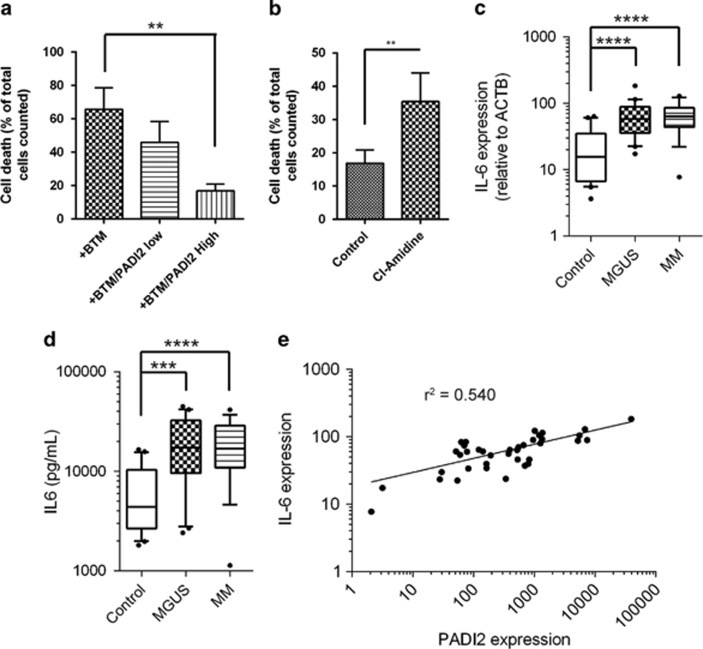
(**a**) Expression of PADI2 protects plasma cells from chemotherapy-induced apoptosis. JJN3 cell apoptosis as a percentage of total cells, either untreated (‘JJN3'), or 24 h after treatment with 10 nm bortezomib (‘+BTM'). When co-cultured with either a low PADI2-expressing (‘PADI2 low') or high PADI2-expressing (‘PADI2 High') BMMSCs across a Boyden chamber, JJN3 cells are partially protected from bortezomib-induced apoptosis, with extent of protection correlating with PADI2 expression. Two primary BMMSC lines were used per assay, each repeated *n*=3. Data were analysed by Kruskal–Wallis test (*P*<0.0001), with Dunn's multiple comparisons test to test for those values that were significantly different. Error bars show s.d. (**b**) Inhibition of total PADI activity in the PADI2 High expressing lines using the pharmacological inhibitor, Cl-Amidine (‘CL') significantly reduces acquired protection from bortezomib. Data were analysed using Mann–Whitney test, error bars show s.d. **P=0.002 (**c**) IL-6 expression is significantly increased in BMMSCs derived from patients with MGUS and MM compared with controls. IL-6 mRNA expression was normalised to beta-actin. Significant changes shown between groups were calculated using Mann–Whitney tests. ****P<0.0001. Error bars indicate 10th-90th percentile (**d**) Bone marrow concentrations of IL-6 are also increased in patients with MGUS and MM. Mann–Whitney tests were again used to calculate significance of differences between groups. ***P=0.0005, ****P<0.0001. (**e**) Scatterplot of the expression of PADI2 against IL-6 as measured in BMMSCs from 38 patients with MM and MGUS shows that their expression is highly correlated (*r*^2^=0.540, non-linear fit).

BMMSCs have been shown to play an important role in the support of malignant plasma cell survival, proliferation and resistance to chemotherapy.^36^ We therefore hypothesised that increased expression of PADI2 may alter the ability of the BMMSC to perform this function. We had previously noted that within the primary BMMSCs we isolated from MGUS and MM patients, some had relatively low expression of PADI2, whereas others were higher. We therefore selected two lines each of ‘low' and ‘high' expressers (Figure 2e, MGUS samples 2 and 5 (low) or 3 and 4 (high), Supplementary Table 2), and co-cultured these with JJN3 cells, a myeloma cell line, using a TransWell system for 24 h before treatment with the clinically relevant chemotherapeutic agent, bortezomib. Although physically separate, the two cell lines could interact through metabolic or secreted factor interactions. We found that although co-culture with low PADI2-expressing BMMSCs had little effect on JJN3 cell viability after bortezomib treatment, high PADI2-expressing BMMSCs resulted in a significant protection, reducing the cell death induced by ~75% (Figure 3a). Interestingly, pre-incubating the high PADI2-expressing BMMSCs with the pan-PADI inhibitor Cl-Amidine before co-culture with JJN3 cells led to a significant increase in bortezomib-induced apoptosis of the JJN3 cells (Figure 3b). These data overall suggested that at least part of the protection from chemotherapy-induced apoptosis elicited by BMMSCs was through a PADI2-mediated event. It had previously been shown that IL-6 production in the bone marrow microenvironment is important for the acquisition of resistance phenotypes by the plasma cells,^20, 37, 38^ with evidence for a role in bortezomib-induced resistance.^39^ We therefore investigated IL-6 expression by BMMSCs, and observed significant increased transcription of IL-6 mRNA (Figure 3c), which led to enhanced secretion into the medium from primary cells from patients with MGUS and MM (Figure 3d). In addition, when mRNA expression of IL-6 was plotted against that of PADI2, we found that they were positively correlated (Figure 3e), providing further evidence for a mechanistic link between these two factors in MGUS and MM.

**Figure 4:**
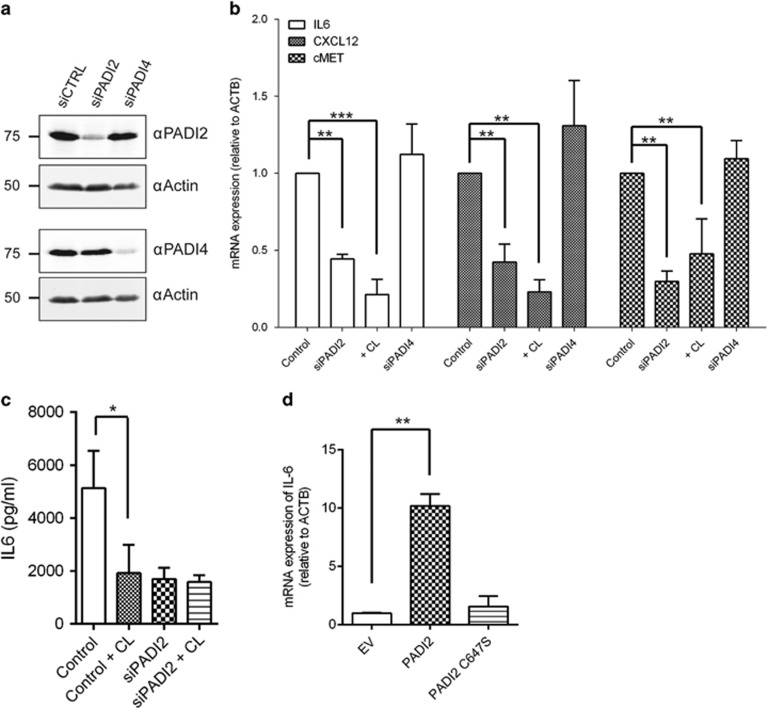
(**a**) Confirmation of knockdown by siPADI2 by two independent siRNA sequences. (**b**) The expression of the BMMSC component of critical chemokine signalling axes in myeloma (IL-6, CXCL12 and cMET) is modulated by PADI2 expression in the immortalised BMMSC cell line, HS5. Expression of all three transcripts are reduced in response to reduction in PADI2 expression (siPADI2), or the addition of the pan-PADI inhibitor, Cl-Amidine (200 μm, 72 h). However, siPADI4 does not alter expression of any of the transcripts. ** and *** indicate the level of significance assigned by a Dunn's comparison *post-test* after a significant ANOVA for each dataset (*P*<0.0001 [IL-6] *P*=0.0002 [CXCL12] *P*=0.0019 [cMET]). (**c**) IL-6 expression, as measured through its secretion into the tissue culture medium by the HS5 cell line, is reduced after treatment with Cl-Amidine and/or siPADI2. Data shown are mean of *n*=3±s.d. *P=0.039, unpaired student's *t*-test with Welch's correction. (**d**) Overexpression of PADI2 increases IL-6 expression, whereas the catalytic mutant, C645S, does not increase expression over basal levels. Data shown are mean of *n*=3±s.d. **P=0.004, unpaired student's *t*-test with Welch's correction.

Using an immortalised, non-transformed BMMSC line (HS5), we assessed the effect of siRNA-mediated reduction in PADI2 expression (Figure 4a) on the expression of IL-6, as well as other factors expressed by BMMSCs known to have a role in MM pathogenesis: CXCL12 and cMET. We found that expression of all three pro-malignant factors was decreased when PADI2 expression was reduced, or PADI activity was inhibited by Cl-Amidine (Figure 4b). Importantly, siRNA of PADI4 (Figure 4a) did not affect expression of any of these factors, showing that this effect was specifically through modulation of PADI2 expression (Figure 4b). Expression of another important factor in the pathogenesis of MM, VEGF, was not found to be modulated by PADI2 or PADI4 expression (Supplementary Figure 3). We directly tested the effect of knocking down or inhibiting PADI2 on IL-6 secretion into the medium of HS5 cells through siRNA or Cl-Amidine treatment, and found that IL-6 was reduced in both conditions, and not further reduced by combining both siRNA and inhibitor (Figure 4c), further supporting our findings that PADI2 is the main PADI family member that regulates IL-6 expression in BMMSCs. Finally, we overexpressed PADI2 and found that IL-6 mRNA expression was increased 10-fold (Figure 4d), whereas expression of an active-site mutant of PADI2, C647S,^40^ had no effect (Figure 4d). These data therefore provide direct mechanistic link between the expression of PADI2 and increased IL-6 production in BMMSCs from MGUS/MM patients.

**Figure 5:**
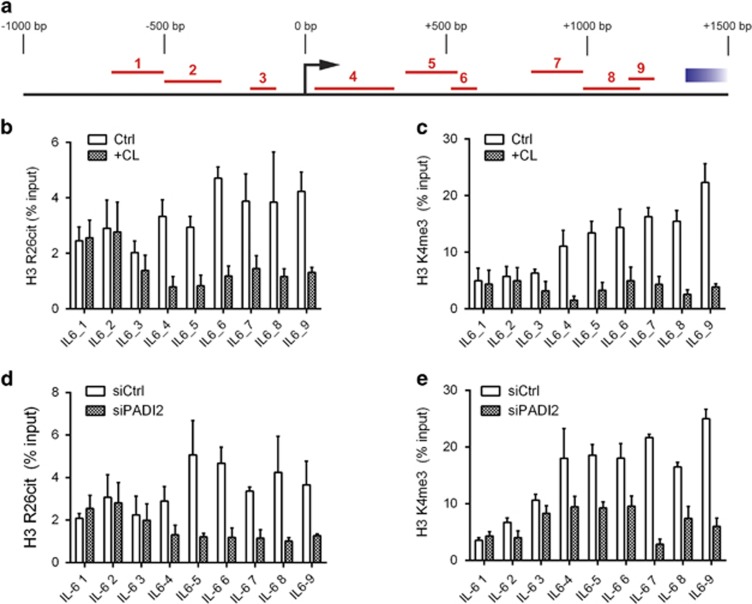
(**a**) Diagram showing the coverage of the *IL-6* promoter region by the primer pairs used in the chromatin immunoprecipitation analysis. Pull-down of chromatin associated with either citrullinated R26 (**b**, **d**) or trimethylated K4 (**c**, **e**) of histone H3, show that both inhibition of PADI activity by Cl-Amidine (**b**, **c**) and reduction of PADI2 expression (through siRNA, **d**, **e**) reduce the enrichment of these marks on and downstream of the *IL-6* transcription start site, indicating that PADI2 activity is required for full activation of transcription from this locus. For all panels, error bars are ±s.e.m.

It has previously been shown that PADI2 activity can citrullinate the R26 residue of histone H3 (H3R26cit), resulting in increased transcription of a number of genes.^27^ Our data also showed increased levels of this modification in BMMSCs from MGUS and MM (Figure 2e). We therefore hypothesised that the mechanism by which PADI2 activity could modulate transcription from the *IL-6* locus was through increasing citrullination of H3R26 in its promoter. Primer pairs that covered the *IL-6* promoter region were designed (Figure 5a), and a chromatin immunoprecipitation experiment performed, in which after either siPADI2 or Cl-Amidine treatment, antibodies against H3R26cit and trimethylated K4 of histone H3 (H3K4me3) were used to pull-down chromatin associated with these modifications. H3K4me3, a modification associated with active areas of chromatin, was used as a positive control to determine whether changes in expression altered the overall activation state of the chromatin. We found that both Cl-Amidine (Figure 5b) and siPADI2 (Figure 5d) decreased the enrichment of H3R26cit in the *IL-6* promoter region, the effects of which were particularly pronounced at and downstream of the transcription start site (TSS; Figure 5a). This correlated with a loss of the activating histone mark, H3K4me3 in the same conditions (Cl-Amidine; Figure 5c, siPADI2; Figure 5e), indicating reduced activation of the *IL-6* promoter locus as a result of reduced PADI2 activity.

**Figure 6:**
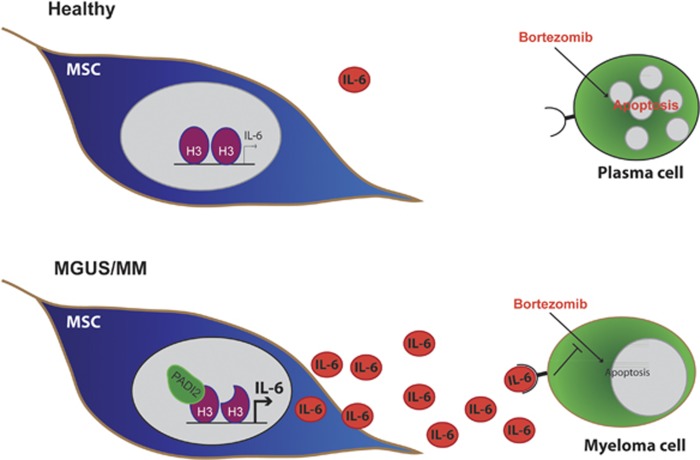
Model of how PADI2 activity in BMMSCs directly contributes to malignant plasma cell phenotype in patients with MGUS and MM. PADI2 citrullinates histone H3 at residue R26 within the promoter region of the *IL-6* gene, which increases its expression and secretion. IL-6 can then mediates its effects on other cells within the bone marrow, including the malignant plasma cells, which become more resistant to chemotherapeutic agents, such as bortezomib.

Our data therefore show that PADI2 expression correlates with IL-6 expression in primary BMMSCs from patients with MGUS and MM (Figure 3) as well as citrullination of the H3R26 (Figure 2e) in the same cells. In addition, we have shown that PADI2 expression can directly increase IL-6 expression (Figure 4) through citrullination of the R26 residue of histone H3 in the promoter region of the gene (Figure 5). Finally, we show that PADI2 expression and activity in BMMSCs can modulate malignant plasma cell resistance to the chemotherapeutic agent, bortezomib (Figures 3a and b, and Figure 6).

## Discussion

MM is an incurable disease that arises from the pre-malignant condition MGUS, present in the bone marrow of up to 7.5% of the elderly population.^1, 41, 42^ With increasing life expectancies and the resulting shift in the demographics of most developed countries, this disease represents a significant clinical challenge. Although it is now clear that the BM microenvironment is transformed in patients with MM, there are few data concerning the nature of the MGUS microenvironment. We demonstrate here that BMMSCs from patients with both MGUS and MM have a transformed transcriptome compared with those from healthy individuals, in agreement with previous results.^7^ Our data, alongside those of others and our previous metabolomic study^35^ highlight the significant similarities between the microenvironment of the BM in MGUS and MM, suggesting that transformation of the BM microenvironment is an early event in disease aetiology, raising the potential of interfering with this process in order to delay or prevent progression of patients with MGUS to MM. In addition, these data further highlight a need to identify those biological determinants in the BM that actually contribute to the progression of this disease: whether these are active drivers, or release of inhibitors of malignant progression.

Our data provide a mechanism by which IL-6 secretion is increased in BMMSCs of patients, which may contribute to treatment failure of bortezomib, a key therapeutic agent in MM treatment strategies. It is important to note, however, that anti-IL-6 therapy has not proven to be an efficacious agent in MM thus far, suggesting that targeting this signalling axis alone is likely to be insufficient to achieve clinical benefit.^43^ PADI2 may therefore represent a novel upstream target, as our data suggest that its activity increases expression of other central signalling networks between BMMSCs and myeloma cells, such as HGF-cMET and CXCL12-CXCR4 (Figure 4b). PADIs are a family of enzymes, of which PADI2 is becoming increasingly linked to a number of chronic diseases, including cancer,^27, 44^ rheumatoid arthritis,^45^ Alzheimer's disease^46^ as well as autoimmune disease.^47^ Targets of the PADI enzymes include proteins with diverse functions such as extracellular matrix formation (for example, vimentin and myelin) and chromatin structure (histone H3).^24^ Importantly, as arginine citrullination is expected to be a relatively long-lived post-translational modification due to the absence of a characterised peptidylcitrullinine iminotransferase, these modifications may only be reversed through protein turnover. In the case of histone H3R26 citrullination within the *IL-6* promoter, this suggests that the chromatin unwinding and activation induced by this modification may only be reversed through proliferation or histone eviction. It is also highly likely that a much larger number of pro-malignant transcripts are also modulated by the activity of PADI2. Indeed, a previous study of the effect of PADI2 on oestrogen receptor-mediated transcriptional regulation showed that it regulated over 200 transcripts in those conditions.^27^ Future work will focus on defining more of these targets in BMMSCs in order to determine the broader implications of PADI2 in the transformation of the healthy BM to that of MGUS/MM.

Therapeutic targeting of PADI2 may therefore represent a good (and early) therapeutic target in MGUS patients as well as those with MM, which may act by removing a significant proportion of the supportive signalling required by malignant plasma cells for survival and proliferation. Indeed, with the growing role for PADI2 in other chronic diseases, this enzyme may represent a novel therapeutic target in many pathological conditions.
