# Supplementary material for: Citrullination of histone H3 drives IL-6 production by bone marrow mesenchymal stem cells in MGUS and multiple myeloma
Source: Leukemia. 2016 Aug 12;31(2):373–81. doi: 10.1038/leu.2016.187 (PMC5292682; doi:10.1038/leu.2016.187)
Supplement: Supplementary Table 1 [file leu2016187x1.docx]

**1a: MM patients**

| **Patient ID** | **Age** | **Sex** | **PP type and level** | **Plasma cells (%)** | **Creatinine**  **(µM)** | **B2M**  **(µg/mL)** | **Albumin**  **(g/L)** | **Stage (ISS)** |
| --- | --- | --- | --- | --- | --- | --- | --- | --- |
| M005 | 72 | M | IgA/k 42g/L | 38% | 80 | 3.2 | 37 | 1 |
| M019 | 71 | M | IgG/l 30g/L | 30% | 133 | 6 | 31 | 3 |
| M023 | 89 | F | IgG/k 32g/L |  | 92 | 6.11 | 34 | 3 |
| M043 | 88 | M | IgA/k 7g/L |  | 236 | N.D. | 36 | N.D. |
| M047 | 55 | M | IgG/k 25g/L | 25-30% | 119 | 5.44 | 38 | 2 |
| M059 | 57 | M | IgG/l 83g/L |  | 110 | 15.1 | 25 | 3 |
| M071 | 68 | F | IgG/l 19g/L | 10-15% | 91 | 3.54 | 39 | 2 |
| M083 | 68 | M | IgA/k 15g/L | >10% | 108 | 3.4 | 38 | 1 |
| M137 | 49 | F | IgG/k 39g/L | 75% | 90 | 3.2 | 35 | 1 |
| M149 | 84 | M | SFLC l4140 |  | 276 | 33.2 | 32 | 3 |

**1b: MGUS patients**

| **Patient ID** | **Age** | **Sex** | **PP type and level** | **Plasma cells (%)** | **sFLC ratio (normal or abnormal)** | **Creatinine**  **(µM)** |
| --- | --- | --- | --- | --- | --- | --- |
| B001 | 51 | F | IgG/L, 4g/L | 3% | Normal | 64 |
| B029 | 52 | F | IgG/L, 2g/L | <1% | Normal | 63 |
| B031 | 71 | M | IgA/L, N.D. | <1% | Normal | 145 |
| B037 | 65 | M | IgG/L, 2g/L | <5% | Normal | 74 |
| B051 | 81 | M | IgG/K, 5g/L | <1% | Normal | 98 |
| B053 | 63 | M | IgG/K, 2g/L | <1% | Abnormal | 100 |
| B055 | 78 | F | IgG/K, 6g/L |  | Abnormal | 65 |
| B073 | 59 | M | IgG/L, 11g/L |  | Abnormal | 92 |
| B095 | 59 | M | IgG/K, 14g/L | 5% | Abnormal | 228 |
| B101 | 75 | M | IgG/L, 12g/L |  | Normal | 206 |
