# Supplementary material for: Citrullination of histone H3 drives IL-6 production by bone marrow mesenchymal stem cells in MGUS and multiple myeloma
Source: Leukemia. 2016 Aug 12;31(2):373–81. doi: 10.1038/leu.2016.187 (PMC5292682; doi:10.1038/leu.2016.187)
Supplement: Supplementary Table 2 [file leu2016187x2.docx]

**MGUS**

| **Patient ID** | **Age** | **Sex** | **PP type and level (g/L)** | **Plasma cells (%)** | **sFLC ratio (normal or abnormal)** | **Creatinine**  **(µM)** | **Figure 2e (sample number)** | **Figure 2f (PADI2 expression)** |
| --- | --- | --- | --- | --- | --- | --- | --- | --- |
| NX007 | 65 | M | IgG/K, 8 | 4 | Abnormal | 114 | 10 |  |
| NX010 | 69 | F | IgG/K, 2 | <1 | Normal | 64 | 9 |  |
| NX043 | 74 | M | IgM/K, 3 | <1 | Abnormal | 148 | 1 |  |
| NX044 | 64 | M | IgA/L, N.D | 9 | Abnormal | 74 | 2 | Low |
| NX046 | 81 | M | IgG/K, <2 | <1 | N.D | 146 | 3 | High |
| NX047 | 78 | M | IgA/K, 3 | 4 | Abnormal | 104 | 4 | High |
| NX048 | 57 | F | IgG/L, 9 | 2 | Abnormal | 67 | 5 | Low |
| NX051 | 56 | F | IgG/L, 3 | 0 | Abnormal | 86 | 6 |  |
| NX052 | 78 | F | IgA/K, <2 | <1 | Abnormal | 84 | 7 |  |
| NX059 | 71 | M | IgG/K, 16 | <5 | Abnormal | 102 | 8 |  |
| NX063 | 64 | M | IgG/L, 8 | 2 | Abnormal | 86 |  |  |
| NX069 | 62 | M | IgG/L, <2 | 0 | Abnormal | 447 |  |  |
| NX070 | 78 | F | IgG/K, 0 | <5 | Abnormal | 338 |  |  |
| NX071 | 81 | M | None (light chain) | 5 | Abnormal | 236 |  |  |
| NX072 | 48 | M | IgA/K, 4 | 4 | Abnormal | 109 |  |  |
| NX073 | 67 | M | IgA/K, 0 | 0 | Abnormal | 72 |  |  |
| NX075 | 62 | M | IgA/K & IgGK, 0 | <1 | Abnormal | 86 |  |  |
| NX076 | 89 | M | IgG/K, 11 | 9 | Abnormal | 114 |  |  |

**MM**

| **Patient ID** | **Age** | **Sex** | **Disease status** | **PP type and level (g/L)** | **Plasma cells (%)** | **Creatinine (µM)** | **B2M**  **(µg/mL)** | **Albumin (g/L)** | **Stage (ISS)** | **Bone lesions** | **Figure**  **2e (sample number)** |
| --- | --- | --- | --- | --- | --- | --- | --- | --- | --- | --- | --- |
| NX045 | 61 | M | New, refractory | IgA/K, <2 | 45 | 567 | 21.8 | 37 | 3 | >2 | 1 |
| NX049 | 70 | M | Relapse | IgA/L, 2 | <1 | 132 | 8.71 | 39 | 3 | >2 | 4 |
| NX050 | 78 | F | New | IgG/K, 17 | 34 | 121 | 4.47 | 33 | 2 | 0 | 5 |
| NX056 | 70 | F | New (response) | IgA/K, N.D | 7 | 138 | 4.46 | 35 | 2 | 0 | 6 |
| NX058 | 72 | F | New | IgM/K, 3 | N.D | 70 | 3.64 | 40 | N.D | N.D | 7 |
| NX060 | 64 | F | New (response) | IgG/K, 36 | 81 | 66 | 3.96 | 33 | 2 | >2 | 8 |
| NX061 | 55 | M | New (response) | IgA/K, 40.6 | 0 | 73 | 4.35 | 32 | 2 | >2 | 9 |
| NX062 | 84 | M | New | IgA/K, 2 | 70 | 93 | 4.13 | 34 | 2 | 0 | 10 |
| NX064 | 81 | M | New | IgG/K, 23 | 13 | 78 | 4.56 | 34 | 2 | 0 |  |
| NX065 | 74 | M | New | IgA/K, 7 | 15-20 | 220 | 5.33 | 35 | 2 | 0 |  |
| NX067 | 45 | M | Refractory | IgG/K, 14 | 6 | 66 | 1.82 | 41 | 1 | 0 |  |
| NX077 | 83 | F | New | IgG/L, 19 | 15 | 61 | 1.9 | 32 | 2 | 0 |  |
| NX078 | 57 | M | New | IgG/K, 18 | 10 | 71 | 2.63 | 24 | 2 | >2 |  |
| NX081 | 84 | M | New (response) | IgA/K, 0 | 0 | 102 | 4.44 | 33 | 1 | 0 |  |
| NX082 | 68 | M | New (response) | IgG/L, 3 | 0 | 81 | 2.18 | 36 | 3 (at diagnosis) | 0 |  |
| NX083 | 68 | M | New | IgG/K, 31 | 18 | 123 | 6.56 | 31 | 3 | >2 |  |
| NX090 | 57 | M | New (response) | IgG/K, <2 | <1 | 74 | 2.49 | 38 | 2 (at diagnosis) | <2 | 2 |
| NX091 | 71 | M | Relapse | IgA/L, <2 | <5 | 158 | 8.87 | 39 | 3 | >2 | 3 |
