# Supplementary material for: Citrullination of histone H3 drives IL-6 production by bone marrow mesenchymal stem cells in MGUS and multiple myeloma
Source: Leukemia. 2016 Aug 12;31(2):373–81. doi: 10.1038/leu.2016.187 (PMC5292682; doi:10.1038/leu.2016.187)
Supplement: Supplementary Table 3 [file leu2016187x3.docx]

| **Target** | **Reference Number** |
| --- | --- |
| ACTB | Hs01060665_g1 |
| IL-6 | Hs00985639_m1 |
| PADI2 | Hs00247108_m1 |
| CXCL12 | Hs03676656_mH |
| cMET | Hs01565576_m1 |
| VEGFA | Hs00900055_m1 |
