# Supplementary material for: Citrullination of histone H3 drives IL-6 production by bone marrow mesenchymal stem cells in MGUS and multiple myeloma
Source: Leukemia. 2016 Aug 12;31(2):373–81. doi: 10.1038/leu.2016.187 (PMC5292682; doi:10.1038/leu.2016.187)
Supplement: Supplementary Table 4 [file leu2016187x4.docx]

| **Primer name** | | **Sequence** |
| --- | --- | --- |
| **1** | F | AAGGGCACAGGTCCTTGATG |
|  | R | ATCAGGGCATGCAGGGAAAA |
| **2** | F | TTTTCCCTGCATGCCCTGAT |
|  | R | CCTGCATGAAACGAAGCCAC |
| **3** | F | ACCACCGTCTCTGTTTAGACAATCG |
|  | R | CGTGCATAACATTTCAGGACCC |
| **4** | F | TGTTGTGCAAGGGTCTGGTT |
|  | R | ATCGCTCCCTCTCCCTGTAA |
| **5** | F | GCCACAAGGTCCTCCTTTGA |
|  | R | GCTCTCTGTGCCAGAGGTTT |
| **6** | F | CTCAGTGGCAATGGGGAGAG |
|  | R | AGTGACCCCTTGGGAATCCT |
| **7** | F | CAGGGAGAGCCAGAACACAG |
|  | R | AGTGACCAGATTAACAGGCTAGA |
| **8** | F | TCTAGCCTGTTAATCTGGTCACT |
|  | R | ACTAGGGGGAAAAGTGCAGC |
| **9** | F | GCTAGCCTCAATGACGACCT |
|  | R | TGGGGCTGATTGGAAACCTT |
