# Supplementary material for: Citrullination of histone H3 drives IL-6 production by bone marrow mesenchymal stem cells in MGUS and multiple myeloma
Source: Leukemia. 2016 Aug 12;31(2):373–81. doi: 10.1038/leu.2016.187 (PMC5292682; doi:10.1038/leu.2016.187)
Supplement: Supplementary Table 5 [file leu2016187x5.docx]

| **Probe Set ID** | **Gene Accession** | **Gene Symbol** | **Gene Description** | **P Value** | **Fold Change** |
| --- | --- | --- | --- | --- | --- |
| 2372103 | NM_005807 | [PRG4](http://www.ncbi.nlm.nih.gov/sites/entrez?db=gene&cmd=&term=PRG4%20%20AND%20Homo) | proteoglycan 4 | 1.19E-04 | -8.61 |
| 2790368 | NM_003013 | [SFRP2](http://www.ncbi.nlm.nih.gov/sites/entrez?db=gene&cmd=&term=SFRP2%20%20AND%20Homo) | secreted frizzled-related protein 2 | 6.52E-06 | -7.77 |
| 3214845 | NM_017680 | [ASPN](http://www.ncbi.nlm.nih.gov/sites/entrez?db=gene&cmd=&term=ASPN%20%20AND%20Homo) | asporin | 1.96E-03 | -5.99 |
| 3359076 | NR_002196 | [H19](http://www.ncbi.nlm.nih.gov/sites/entrez?db=gene&cmd=&term=H19%20%20AND%20Homo) | H19, imprinted maternally expressed transcript (non-protein coding) | 5.70E-06 | -5.82 |
| 2863363 | NM_004101 | [F2RL2](http://www.ncbi.nlm.nih.gov/sites/entrez?db=gene&cmd=&term=F2RL2%20%20AND%20Homo) | coagulation factor II (thrombin) receptor-like 2 | 1.52E-03 | -5.77 |
| 3321361 | NM_006108 | [SPON1](http://www.ncbi.nlm.nih.gov/sites/entrez?db=gene&cmd=&term=SPON1%20%20AND%20Homo) | spondin 1, extracellular matrix protein | 4.82E-04 | -4.59 |
| 2816459 | NM_001992 | [F2R](http://www.ncbi.nlm.nih.gov/sites/entrez?db=gene&cmd=&term=F2R%20%20AND%20Homo) | coagulation factor II (thrombin) receptor | 4.72E-06 | -4.23 |
| 3013255 | NM_001040152 | [PEG10](http://www.ncbi.nlm.nih.gov/sites/entrez?db=gene&cmd=&term=PEG10%20%20AND%20Homo) | paternally expressed 10 | 4.08E-04 | -3.94 |
| 2905296 | NM_153370 | [PI16](http://www.ncbi.nlm.nih.gov/sites/entrez?db=gene&cmd=&term=PI16%20%20AND%20Homo) | peptidase inhibitor 16 | 1.20E-04 | -3.90 |
| 4018080 | NM_145234 | [CHRDL1](http://www.ncbi.nlm.nih.gov/sites/entrez?db=gene&cmd=&term=CHRDL1%20%20AND%20Homo) | chordin-like 1 | 9.21E-04 | -3.45 |
| 3046444 | NM_003014 | [SFRP4](http://www.ncbi.nlm.nih.gov/sites/entrez?db=gene&cmd=&term=SFRP4%20%20AND%20Homo) | secreted frizzled-related protein 4 | 7.22E-04 | -3.44 |
| 3605395 | NM_207517 | [ADAMTSL3](http://www.ncbi.nlm.nih.gov/sites/entrez?db=gene&cmd=&term=ADAMTSL3%20%20AND%20Homo) | ADAMTS-like 3 | 1.12E-04 | -3.27 |
| 3783529 | NM_001943 | [DSG2](http://www.ncbi.nlm.nih.gov/sites/entrez?db=gene&cmd=&term=DSG2%20%20AND%20Homo) | desmoglein 2 | 6.23E-04 | -3.11 |
| 3214800 | NM_033014 | [OGN](http://www.ncbi.nlm.nih.gov/sites/entrez?db=gene&cmd=&term=OGN%20%20AND%20Homo) | osteoglycin | 6.60E-03 | -3.00 |
| 3416353 | NM_022658 | [HOXC8](http://www.ncbi.nlm.nih.gov/sites/entrez?db=gene&cmd=&term=HOXC8%20%20AND%20Homo) | homeobox C8 | 1.67E-09 | -2.95 |
| 2644202 | NM_144717 | [IL20RB](http://www.ncbi.nlm.nih.gov/sites/entrez?db=gene&cmd=&term=IL20RB%20%20AND%20Homo) | interleukin 20 receptor beta | 4.70E-03 | -2.91 |
| 3389077 | NM_025208 | [PDGFD](http://www.ncbi.nlm.nih.gov/sites/entrez?db=gene&cmd=&term=PDGFD%20%20AND%20Homo) | platelet derived growth factor D | 1.66E-04 | -2.88 |
| 3057955 | NM_006682 | [FGL2](http://www.ncbi.nlm.nih.gov/sites/entrez?db=gene&cmd=&term=FGL2%20%20AND%20Homo) | fibrinogen-like 2 | 2.64E-03 | -2.80 |
| 3087703 | NM_006207 | [PDGFRL](http://www.ncbi.nlm.nih.gov/sites/entrez?db=gene&cmd=&term=PDGFRL%20%20AND%20Homo) | platelet-derived growth factor receptor-like | 2.96E-05 | -2.65 |
| 3420487 | NM_007199 | [IRAK3](http://www.ncbi.nlm.nih.gov/sites/entrez?db=gene&cmd=&term=IRAK3%20%20AND%20Homo) | interleukin-1 receptor-associated kinase 3 | 2.17E-04 | -2.61 |
| 3310953 | NM_198148 | [CPXM2](http://www.ncbi.nlm.nih.gov/sites/entrez?db=gene&cmd=&term=CPXM2%20%20AND%20Homo) | carboxypeptidase X (M14 family), member 2 | 1.85E-03 | -2.53 |
| 2357040 | AK023809 | [LOC284561](http://www.ncbi.nlm.nih.gov/sites/entrez?db=gene&cmd=&term=LOC284561%20%20AND%20Homo) | hypothetical protein LOC284561 | 8.44E-04 | -2.52 |
| 3446919 | NM_020297 | [ABCC9](http://www.ncbi.nlm.nih.gov/sites/entrez?db=gene&cmd=&term=ABCC9%20%20AND%20Homo) | ATP-binding cassette, sub-family C (CFTR/MRP), member 9 | 5.08E-04 | -2.51 |
| 3801943 | NM_015461 | [ZNF521](http://www.ncbi.nlm.nih.gov/sites/entrez?db=gene&cmd=&term=ZNF521%20%20AND%20Homo) | zinc finger protein 521 | 6.03E-05 | -2.37 |
| 2451693 | NM_002023 | [FMOD](http://www.ncbi.nlm.nih.gov/sites/entrez?db=gene&cmd=&term=FMOD%20%20AND%20Homo) | fibromodulin | 1.33E-04 | -2.35 |
| 3438061 | NM_198827 | [GPR133](http://www.ncbi.nlm.nih.gov/sites/entrez?db=gene&cmd=&term=GPR133%20%20AND%20Homo) | G protein-coupled receptor 133 | 5.66E-05 | -2.32 |
| 3901041 | NM_000361 | [THBD](http://www.ncbi.nlm.nih.gov/sites/entrez?db=gene&cmd=&term=THBD%20%20AND%20Homo) | thrombomodulin | 1.55E-04 | -2.31 |
| 3214825 | NM_005014 | [OMD](http://www.ncbi.nlm.nih.gov/sites/entrez?db=gene&cmd=&term=OMD%20%20AND%20Homo) | osteomodulin | 3.27E-03 | -2.29 |
| 2375680 | XM_002342128 | [LOC100288349](http://www.ncbi.nlm.nih.gov/sites/entrez?db=gene&cmd=&term=LOC100288349%20%20AND%20Homo) | hypothetical protein LOC100288349 | 2.61E-05 | -2.25 |
| 3855104 | NM_004750 | [CRLF1](http://www.ncbi.nlm.nih.gov/sites/entrez?db=gene&cmd=&term=CRLF1%20%20AND%20Homo) | cytokine receptor-like factor 1 | 2.74E-03 | -2.24 |
| 2925237 | NM_000426 | [LAMA2](http://www.ncbi.nlm.nih.gov/sites/entrez?db=gene&cmd=&term=LAMA2%20%20AND%20Homo) | laminin, alpha 2 | 8.68E-04 | -2.19 |
| 3761441 | NM_024016 | [HOXB8](http://www.ncbi.nlm.nih.gov/sites/entrez?db=gene&cmd=&term=HOXB8%20%20AND%20Homo) | homeobox B8 | 3.82E-09 | -2.17 |
| 3445741 | NM_000900 | [MGP](http://www.ncbi.nlm.nih.gov/sites/entrez?db=gene&cmd=&term=MGP%20%20AND%20Homo) | matrix Gla protein | 7.92E-03 | -2.14 |
| 3768535 | NM_017565 | [FAM20A](http://www.ncbi.nlm.nih.gov/sites/entrez?db=gene&cmd=&term=FAM20A%20%20AND%20Homo) | family with sequence similarity 20, member A | 2.43E-04 | -2.12 |
| 3319119 | NM_198474 | [OLFML1](http://www.ncbi.nlm.nih.gov/sites/entrez?db=gene&cmd=&term=OLFML1%20%20AND%20Homo) | olfactomedin-like 1 | 8.59E-03 | -2.12 |
| 3059942 | NM_001142749 | [KIAA1324L](http://www.ncbi.nlm.nih.gov/sites/entrez?db=gene&cmd=&term=KIAA1324L%20%20AND%20Homo) | KIAA1324-like | 4.44E-03 | -2.12 |
| 3761311 | NM_002146 | [HOXB3](http://www.ncbi.nlm.nih.gov/sites/entrez?db=gene&cmd=&term=HOXB3%20%20AND%20Homo) | homeobox B3 | 5.23E-06 | -2.11 |
| 3748798 | NM_002404 | [MFAP4](http://www.ncbi.nlm.nih.gov/sites/entrez?db=gene&cmd=&term=MFAP4%20%20AND%20Homo) | microfibrillar-associated protein 4 | 2.60E-04 | -2.11 |
| 3132782 | NM_003012 | [SFRP1](http://www.ncbi.nlm.nih.gov/sites/entrez?db=gene&cmd=&term=SFRP1%20%20AND%20Homo) | secreted frizzled-related protein 1 | 8.62E-03 | -2.10 |
| 3574121 | NM_033104 | [STON2](http://www.ncbi.nlm.nih.gov/sites/entrez?db=gene&cmd=&term=STON2%20%20AND%20Homo) | stonin 2 | 1.08E-04 | -2.08 |
| 2758870 | NM_018659 | [CYTL1](http://www.ncbi.nlm.nih.gov/sites/entrez?db=gene&cmd=&term=CYTL1%20%20AND%20Homo) | cytokine-like 1 | 4.28E-04 | -2.07 |
| 3198346 | NM_002839 | [PTPRD](http://www.ncbi.nlm.nih.gov/sites/entrez?db=gene&cmd=&term=PTPRD%20%20AND%20Homo) | protein tyrosine phosphatase, receptor type, D | 1.08E-03 | -2.06 |
| 2445982 | NM_004673 | [ANGPTL1](http://www.ncbi.nlm.nih.gov/sites/entrez?db=gene&cmd=&term=ANGPTL1%20%20AND%20Homo) | angiopoietin-like 1 | 3.54E-03 | -2.04 |
| 3705967 | NM_002615 | [SERPINF1](http://www.ncbi.nlm.nih.gov/sites/entrez?db=gene&cmd=&term=SERPINF1%20%20AND%20Homo) | serpin peptidase inhibitor, clade F (alpha-2 antiplasmin, pigment epithelium derived factor), member 1 | 1.99E-05 | -2.03 |
| 3956226 | NM_002430 | [MN1](http://www.ncbi.nlm.nih.gov/sites/entrez?db=gene&cmd=&term=MN1%20%20AND%20Homo) | meningioma (disrupted in balanced translocation) 1 | 7.36E-04 | -2.01 |
| 3488592 | NM_005694 | [COX17](http://www.ncbi.nlm.nih.gov/sites/entrez?db=gene&cmd=&term=COX17%20%20AND%20Homo) | COX17 cytochrome c oxidase assembly homolog (S. cerevisiae) | 1.33E-03 | 2.00 |
| 2412624 | NM_002867 | [RAB3B](http://www.ncbi.nlm.nih.gov/sites/entrez?db=gene&cmd=&term=RAB3B%20%20AND%20Homo) | RAB3B, member RAS oncogene family | 4.09E-03 | 2.00 |
| 3266408 | NM_004098 | [EMX2](http://www.ncbi.nlm.nih.gov/sites/entrez?db=gene&cmd=&term=EMX2%20%20AND%20Homo) | empty spiracles homeobox 2 | 3.42E-06 | 2.00 |
| 3436329 | NM_181709 | [FAM101A](http://www.ncbi.nlm.nih.gov/sites/entrez?db=gene&cmd=&term=FAM101A%20%20AND%20Homo) | family with sequence similarity 101, member A | 3.13E-04 | 2.01 |
| 2809245 | NM_002203 | [ITGA2](http://www.ncbi.nlm.nih.gov/sites/entrez?db=gene&cmd=&term=ITGA2%20%20AND%20Homo) | integrin, alpha 2 (CD49B, alpha 2 subunit of VLA-2 receptor) | 1.65E-03 | 2.01 |
| 2704998 | Z19588 | [SKIL](http://www.ncbi.nlm.nih.gov/sites/entrez?db=gene&cmd=&term=SKIL%20%20AND%20Homo) | SKI-like oncogene | 6.94E-05 | 2.05 |
| 2749472 | XR_015691 | [LOC646890](http://www.ncbi.nlm.nih.gov/sites/entrez?db=gene&cmd=&term=LOC646890%20%20AND%20Homo) | hypothetical LOC646890 | 4.55E-03 | 2.08 |
| 3049522 | NM_022748 | [TNS3](http://www.ncbi.nlm.nih.gov/sites/entrez?db=gene&cmd=&term=TNS3%20%20AND%20Homo) | tensin 3 | 2.16E-04 | 2.08 |
| 3631397 | NM_018003 | [UACA](http://www.ncbi.nlm.nih.gov/sites/entrez?db=gene&cmd=&term=UACA%20%20AND%20Homo) | uveal autoantigen with coiled-coil domains and ankyrin repeats | 1.22E-04 | 2.08 |
| 3756723 | NM_033184 | [KRTAP2-4](http://www.ncbi.nlm.nih.gov/sites/entrez?db=gene&cmd=&term=KRTAP2-4%20%20AND%20Homo) | keratin associated protein 2-4 | 3.25E-03 | 2.14 |
| 3402786 | NM_000616 | [CD4](http://www.ncbi.nlm.nih.gov/sites/entrez?db=gene&cmd=&term=CD4%20%20AND%20Homo) | CD4 molecule | 8.16E-04 | 2.15 |
| 2926323 | NM_004100 | [EYA4](http://www.ncbi.nlm.nih.gov/sites/entrez?db=gene&cmd=&term=EYA4%20%20AND%20Homo) | eyes absent homolog 4 (Drosophila) | 2.43E-04 | 2.16 |
| 3150455 | NM_002546 | [TNFRSF11B](http://www.ncbi.nlm.nih.gov/sites/entrez?db=gene&cmd=&term=TNFRSF11B%20%20AND%20Homo) | tumor necrosis factor receptor superfamily, member 11b | 5.73E-03 | 2.20 |
| 2710474 | NM_018192 | [LEPREL1](http://www.ncbi.nlm.nih.gov/sites/entrez?db=gene&cmd=&term=LEPREL1%20%20AND%20Homo) | leprecan-like 1 | 1.65E-04 | 2.21 |
| 3982023 | NM_016120 | [RLIM](http://www.ncbi.nlm.nih.gov/sites/entrez?db=gene&cmd=&term=RLIM%20%20AND%20Homo) | ring finger protein, LIM domain interacting | 1.06E-04 | 2.22 |
| 3550307 | NM_000710 | [BDKRB1](http://www.ncbi.nlm.nih.gov/sites/entrez?db=gene&cmd=&term=BDKRB1%20%20AND%20Homo) | bradykinin receptor B1 | 3.99E-03 | 2.32 |
| 2509988 | NM_177964 | [LYPD6B](http://www.ncbi.nlm.nih.gov/sites/entrez?db=gene&cmd=&term=LYPD6B%20%20AND%20Homo) | LY6/PLAUR domain containing 6B | 3.74E-04 | 2.33 |
| 2783207 | NM_003619 | [PRSS12](http://www.ncbi.nlm.nih.gov/sites/entrez?db=gene&cmd=&term=PRSS12%20%20AND%20Homo) | protease, serine, 12 (neurotrypsin, motopsin) | 8.54E-05 | 2.33 |
| 3042919 | NM_152739 | [HOXA9](http://www.ncbi.nlm.nih.gov/sites/entrez?db=gene&cmd=&term=HOXA9%20%20AND%20Homo) | homeobox A9 | 9.21E-10 | 2.37 |
| 2970525 | BC037331 | [LOC285758](http://www.ncbi.nlm.nih.gov/sites/entrez?db=gene&cmd=&term=LOC285758%20%20AND%20Homo) | hypothetical protein LOC285758 | 1.13E-04 | 2.37 |
| 2614798 | NM_001145160 | [TPM4](http://www.ncbi.nlm.nih.gov/sites/entrez?db=gene&cmd=&term=TPM4%20%20AND%20Homo) | tropomyosin 4 | 9.98E-03 | 2.39 |
| 3452478 | NM_001143668 | [AMIGO2](http://www.ncbi.nlm.nih.gov/sites/entrez?db=gene&cmd=&term=AMIGO2%20%20AND%20Homo) | adhesion molecule with Ig-like domain 2 | 7.12E-03 | 2.41 |
| 2893794 | NM_004415 | [DSP](http://www.ncbi.nlm.nih.gov/sites/entrez?db=gene&cmd=&term=DSP%20%20AND%20Homo) | desmoplakin | 1.67E-07 | 2.52 |
| 3394264 | NM_006500 | [MCAM](http://www.ncbi.nlm.nih.gov/sites/entrez?db=gene&cmd=&term=MCAM%20%20AND%20Homo) | melanoma cell adhesion molecule | 4.20E-04 | 2.56 |
| 3631394 | NM_018003 | [UACA](http://www.ncbi.nlm.nih.gov/sites/entrez?db=gene&cmd=&term=UACA%20%20AND%20Homo) | uveal autoantigen with coiled-coil domains and ankyrin repeats | 2.47E-04 | 2.65 |
| 3023883 | NM_016352 | [CPA4](http://www.ncbi.nlm.nih.gov/sites/entrez?db=gene&cmd=&term=CPA4%20%20AND%20Homo) | carboxypeptidase A4 | 3.78E-07 | 2.65 |
| 2364381 | NM_001102445 | [RGS4](http://www.ncbi.nlm.nih.gov/sites/entrez?db=gene&cmd=&term=RGS4%20%20AND%20Homo) | regulator of G-protein signaling 4 | 3.69E-04 | 2.66 |
| 2527253 | NM_000597 | [IGFBP2](http://www.ncbi.nlm.nih.gov/sites/entrez?db=gene&cmd=&term=IGFBP2%20%20AND%20Homo) | insulin-like growth factor binding protein 2, 36kDa | 6.36E-03 | 2.67 |
| 3930525 | NR_026812 | [C21orf96](http://www.ncbi.nlm.nih.gov/sites/entrez?db=gene&cmd=&term=C21orf96%20%20AND%20Homo) | chromosome 21 open reading frame 96 | 7.09E-06 | 2.68 |
| 3262129 | NM_032727 | [INA](http://www.ncbi.nlm.nih.gov/sites/entrez?db=gene&cmd=&term=INA%20%20AND%20Homo) | internexin neuronal intermediate filament protein, alpha | 2.43E-03 | 2.73 |
| 3299970 | NM_014391 | [ANKRD1](http://www.ncbi.nlm.nih.gov/sites/entrez?db=gene&cmd=&term=ANKRD1%20%20AND%20Homo) | ankyrin repeat domain 1 (cardiac muscle) | 1.35E-03 | 2.75 |
| 2590715 | NM_001463 | [FRZB](http://www.ncbi.nlm.nih.gov/sites/entrez?db=gene&cmd=&term=FRZB%20%20AND%20Homo) | frizzled-related protein | 3.21E-03 | 2.76 |
| 3905145 | NM_004613 | [TGM2](http://www.ncbi.nlm.nih.gov/sites/entrez?db=gene&cmd=&term=TGM2%20%20AND%20Homo) | transglutaminase 2 (C polypeptide, protein-glutamine-gamma-glutamyltransferase) | 3.28E-04 | 2.79 |
| 2848257 | AK124354 | [CCT5](http://www.ncbi.nlm.nih.gov/sites/entrez?db=gene&cmd=&term=CCT5%20%20AND%20Homo) | chaperonin containing TCP1, subunit 5 (epsilon) | 1.35E-06 | 2.84 |
| 3063035 | NM_001134450 | [TMEM130](http://www.ncbi.nlm.nih.gov/sites/entrez?db=gene&cmd=&term=TMEM130%20%20AND%20Homo) | transmembrane protein 130 | 5.76E-05 | 2.92 |
| 3416290 | NM_017409 | [HOXC10](http://www.ncbi.nlm.nih.gov/sites/entrez?db=gene&cmd=&term=HOXC10%20%20AND%20Homo) | homeobox C10 | 5.62E-08 | 2.92 |
| 2805078 | NM_004932 | [CDH6](http://www.ncbi.nlm.nih.gov/sites/entrez?db=gene&cmd=&term=CDH6%20%20AND%20Homo) | cadherin 6, type 2, K-cadherin (fetal kidney) | 3.64E-05 | 2.95 |
| 3277110 | NR_027082 | [SFTA1P](http://www.ncbi.nlm.nih.gov/sites/entrez?db=gene&cmd=&term=SFTA1P%20%20AND%20Homo) | surfactant associated 1 (pseudogene) | 7.91E-03 | 3.02 |
| 2731496 | NM_001013442 | [EPGN](http://www.ncbi.nlm.nih.gov/sites/entrez?db=gene&cmd=&term=EPGN%20%20AND%20Homo) | epithelial mitogen homolog (mouse) | 1.00E-03 | 3.04 |
| 2743800 | NM_032961 | [PCDH10](http://www.ncbi.nlm.nih.gov/sites/entrez?db=gene&cmd=&term=PCDH10%20%20AND%20Homo) | protocadherin 10 | 7.49E-04 | 3.10 |
| 3756689 | NM_030967 | [KRTAP1-1](http://www.ncbi.nlm.nih.gov/sites/entrez?db=gene&cmd=&term=KRTAP1-1%20%20AND%20Homo) | keratin associated protein 1-1 | 1.35E-06 | 3.21 |
| 3726154 | NM_002204 | [ITGA3](http://www.ncbi.nlm.nih.gov/sites/entrez?db=gene&cmd=&term=ITGA3%20%20AND%20Homo) | integrin, alpha 3 (antigen CD49C, alpha 3 subunit of VLA-3 receptor) | 1.92E-06 | 3.22 |
| 3756676 | NM_031957 // NM_031957 // NM_031957 | [KRTAP1-5 // KRTAP1-5 // KRTAP1-5](http://www.ncbi.nlm.nih.gov/sites/entrez?db=gene&cmd=&term=KRTAP1-5%20%20//%20KRTAP1-5%20%20//%20KRTAP1-5%20%20AND%20Homo) | keratin associated protein 1-5 // keratin associated protein 1-5 // keratin associated protein 1-5 | 6.58E-05 | 3.64 |
| 2990404 | NM_001112706 | [SCIN](http://www.ncbi.nlm.nih.gov/sites/entrez?db=gene&cmd=&term=SCIN%20%20AND%20Homo) | scinderin | 1.03E-05 | 3.93 |
| 3786868 | NM_001128588 | [SLC14A1](http://www.ncbi.nlm.nih.gov/sites/entrez?db=gene&cmd=&term=SLC14A1%20%20AND%20Homo) | solute carrier family 14 (urea transporter), member 1 (Kidd blood group) | 6.80E-06 | 5.47 |
| 2435649 | NM_002016 | [FLG](http://www.ncbi.nlm.nih.gov/sites/entrez?db=gene&cmd=&term=FLG%20%20AND%20Homo) | filaggrin | 2.71E-04 | 6.39 |
