# Supplementary material for: Citrullination of histone H3 drives IL-6 production by bone marrow mesenchymal stem cells in MGUS and multiple myeloma
Source: Leukemia. 2016 Aug 12;31(2):373–81. doi: 10.1038/leu.2016.187 (PMC5292682; doi:10.1038/leu.2016.187)
Supplement: Supplementary Table 6 [file leu2016187x6.docx]

| **Probe Set ID** | **Gene Accession** | **Gene Symbol** | **Gene Description** | **P.Value** | **Fold Change** |
| --- | --- | --- | --- | --- | --- |
| 2372103 | NM_005807 | [PRG4](http://www.ncbi.nlm.nih.gov/sites/entrez?db=gene&cmd=&term=PRG4%20%20AND%20Homo) | proteoglycan 4 | 1.36E-04 | -8.41 |
| 3214845 | NM_017680 | [ASPN](http://www.ncbi.nlm.nih.gov/sites/entrez?db=gene&cmd=&term=ASPN%20%20AND%20Homo) | asporin | 1.28E-03 | -5.96 |
| 2863363 | NM_004101 | [F2RL2](http://www.ncbi.nlm.nih.gov/sites/entrez?db=gene&cmd=&term=F2RL2%20%20AND%20Homo) | coagulation factor II (thrombin) receptor-like 2 | 5.37E-04 | -5.58 |
| 3359076 | NR_002196 | [H19](http://www.ncbi.nlm.nih.gov/sites/entrez?db=gene&cmd=&term=H19%20%20AND%20Homo) | H19, imprinted maternally expressed transcript (non-protein coding) | 1.38E-05 | -5.34 |
| 3321361 | NM_006108 | [SPON1](http://www.ncbi.nlm.nih.gov/sites/entrez?db=gene&cmd=&term=SPON1%20%20AND%20Homo) | spondin 1, extracellular matrix protein | 2.91E-04 | -5.02 |
| 2790368 | NM_003013 | [SFRP2](http://www.ncbi.nlm.nih.gov/sites/entrez?db=gene&cmd=&term=SFRP2%20%20AND%20Homo) | secreted frizzled-related protein 2 | 7.33E-05 | -4.72 |
| 2816459 | NM_001992 | [F2R](http://www.ncbi.nlm.nih.gov/sites/entrez?db=gene&cmd=&term=F2R%20%20AND%20Homo) | coagulation factor II (thrombin) receptor | 9.38E-06 | -4.54 |
| 3104489 | NM_007029 | [STMN2](http://www.ncbi.nlm.nih.gov/sites/entrez?db=gene&cmd=&term=STMN2%20%20AND%20Homo) | stathmin-like 2 | 9.18E-04 | -4.48 |
| 2905296 | NM_153370 | [PI16](http://www.ncbi.nlm.nih.gov/sites/entrez?db=gene&cmd=&term=PI16%20%20AND%20Homo) | peptidase inhibitor 16 | 5.68E-05 | -4.23 |
| 3605395 | NM_207517 | [ADAMTSL3](http://www.ncbi.nlm.nih.gov/sites/entrez?db=gene&cmd=&term=ADAMTSL3%20%20AND%20Homo) | ADAMTS-like 3 | 1.09E-05 | -4.02 |
| 3046444 | NM_003014 | [SFRP4](http://www.ncbi.nlm.nih.gov/sites/entrez?db=gene&cmd=&term=SFRP4%20%20AND%20Homo) | secreted frizzled-related protein 4 | 3.36E-05 | -4.01 |
| 3388807 | NM_002421 | [MMP1](http://www.ncbi.nlm.nih.gov/sites/entrez?db=gene&cmd=&term=MMP1%20%20AND%20Homo) | matrix metallopeptidase 1 (interstitial collagenase) | 2.82E-03 | -3.93 |
| 4018729 | NM_000640 | [IL13RA2](http://www.ncbi.nlm.nih.gov/sites/entrez?db=gene&cmd=&term=IL13RA2%20%20AND%20Homo) | interleukin 13 receptor, alpha 2 | 2.92E-03 | -3.32 |
| 3783529 | NM_001943 | [DSG2](http://www.ncbi.nlm.nih.gov/sites/entrez?db=gene&cmd=&term=DSG2%20%20AND%20Homo) | desmoglein 2 | 7.77E-04 | -3.05 |
| 3013255 | NM_001040152 | [PEG10](http://www.ncbi.nlm.nih.gov/sites/entrez?db=gene&cmd=&term=PEG10%20%20AND%20Homo) | paternally expressed 10 | 3.11E-03 | -2.94 |
| 3389077 | NM_025208 | [PDGFD](http://www.ncbi.nlm.nih.gov/sites/entrez?db=gene&cmd=&term=PDGFD%20%20AND%20Homo) | platelet derived growth factor D | 6.98E-04 | -2.93 |
| 3128411 | NM_022659 | [EBF2](http://www.ncbi.nlm.nih.gov/sites/entrez?db=gene&cmd=&term=EBF2%20%20AND%20Homo) | early B-cell factor 2 | 9.09E-03 | -2.91 |
| 3214800 | NM_033014 | [OGN](http://www.ncbi.nlm.nih.gov/sites/entrez?db=gene&cmd=&term=OGN%20%20AND%20Homo) | osteoglycin | 8.60E-03 | -2.87 |
| 4018080 | NM_145234 | [CHRDL1](http://www.ncbi.nlm.nih.gov/sites/entrez?db=gene&cmd=&term=CHRDL1%20%20AND%20Homo) | chordin-like 1 | 1.35E-03 | -2.79 |
| 3021009 | NM_012281 | [KCND2](http://www.ncbi.nlm.nih.gov/sites/entrez?db=gene&cmd=&term=KCND2%20%20AND%20Homo) | potassium voltage-gated channel, Shal-related subfamily, member 2 | 7.48E-04 | -2.65 |
| 2357040 | AK023809 | [LOC284561](http://www.ncbi.nlm.nih.gov/sites/entrez?db=gene&cmd=&term=LOC284561%20%20AND%20Homo) | hypothetical protein LOC284561 | 3.64E-04 | -2.53 |
| 3446919 | NM_020297 | [ABCC9](http://www.ncbi.nlm.nih.gov/sites/entrez?db=gene&cmd=&term=ABCC9%20%20AND%20Homo) | ATP-binding cassette, sub-family C (CFTR/MRP), member 9 | 1.39E-03 | -2.53 |
| 3057955 | NM_006682 | [FGL2](http://www.ncbi.nlm.nih.gov/sites/entrez?db=gene&cmd=&term=FGL2%20%20AND%20Homo) | fibrinogen-like 2 | 6.05E-03 | -2.52 |
| 3438061 | NM_198827 | [GPR133](http://www.ncbi.nlm.nih.gov/sites/entrez?db=gene&cmd=&term=GPR133%20%20AND%20Homo) | G protein-coupled receptor 133 | 7.90E-07 | -2.44 |
| 3416353 | NM_022658 | [HOXC8](http://www.ncbi.nlm.nih.gov/sites/entrez?db=gene&cmd=&term=HOXC8%20%20AND%20Homo) | homeobox C8 | 5.57E-07 | -2.43 |
| 3420487 | NM_007199 | [IRAK3](http://www.ncbi.nlm.nih.gov/sites/entrez?db=gene&cmd=&term=IRAK3%20%20AND%20Homo) | interleukin-1 receptor-associated kinase 3 | 5.43E-06 | -2.36 |
| 2368590 | NM_020318 | [PAPPA2](http://www.ncbi.nlm.nih.gov/sites/entrez?db=gene&cmd=&term=PAPPA2%20%20AND%20Homo) | pappalysin 2 | 4.24E-06 | -2.27 |
| 3087703 | NM_006207 | [PDGFRL](http://www.ncbi.nlm.nih.gov/sites/entrez?db=gene&cmd=&term=PDGFRL%20%20AND%20Homo) | platelet-derived growth factor receptor-like | 1.34E-03 | -2.23 |
| 3768703 | NM_080283 | [ABCA9](http://www.ncbi.nlm.nih.gov/sites/entrez?db=gene&cmd=&term=ABCA9%20%20AND%20Homo) | ATP-binding cassette, sub-family A (ABC1), member 9 | 3.41E-05 | -2.23 |
| 3214825 | NM_005014 | [OMD](http://www.ncbi.nlm.nih.gov/sites/entrez?db=gene&cmd=&term=OMD%20%20AND%20Homo) | osteomodulin | 1.92E-03 | -2.23 |
| 3761441 | NM_024016 | [HOXB8](http://www.ncbi.nlm.nih.gov/sites/entrez?db=gene&cmd=&term=HOXB8%20%20AND%20Homo) | homeobox B8 | 4.12E-09 | -2.22 |
| 3855104 | NM_004750 | [CRLF1](http://www.ncbi.nlm.nih.gov/sites/entrez?db=gene&cmd=&term=CRLF1%20%20AND%20Homo) | cytokine receptor-like factor 1 | 2.97E-03 | -2.21 |
| 3574121 | NM_033104 | [STON2](http://www.ncbi.nlm.nih.gov/sites/entrez?db=gene&cmd=&term=STON2%20%20AND%20Homo) | stonin 2 | 1.12E-03 | -2.16 |
| 2375680 | XM_002342128 | [LOC100288349](http://www.ncbi.nlm.nih.gov/sites/entrez?db=gene&cmd=&term=LOC100288349%20%20AND%20Homo) | hypothetical protein LOC100288349 | 1.02E-03 | -2.15 |
| 3132782 | NM_003012 | [SFRP1](http://www.ncbi.nlm.nih.gov/sites/entrez?db=gene&cmd=&term=SFRP1%20%20AND%20Homo) | secreted frizzled-related protein 1 | 2.10E-03 | -2.10 |
| 3901041 | NM_000361 | [THBD](http://www.ncbi.nlm.nih.gov/sites/entrez?db=gene&cmd=&term=THBD%20%20AND%20Homo) | thrombomodulin | 5.41E-04 | -2.09 |
| 3059942 | NM_001142749 | [KIAA1324L](http://www.ncbi.nlm.nih.gov/sites/entrez?db=gene&cmd=&term=KIAA1324L%20%20AND%20Homo) | KIAA1324-like | 4.05E-03 | -2.09 |
| 3181600 | NM_024642 | [GALNT12](http://www.ncbi.nlm.nih.gov/sites/entrez?db=gene&cmd=&term=GALNT12%20%20AND%20Homo) | UDP-N-acetyl-alpha-D-galactosamine:polypeptide N-acetylgalactosaminyltransferase 12 (GalNAc-T12) | 6.16E-03 | -2.06 |
| 3768535 | NM_017565 | [FAM20A](http://www.ncbi.nlm.nih.gov/sites/entrez?db=gene&cmd=&term=FAM20A%20%20AND%20Homo) | family with sequence similarity 20, member A | 7.94E-04 | -2.04 |
| 3461121 | NM_018402 | [IL26](http://www.ncbi.nlm.nih.gov/sites/entrez?db=gene&cmd=&term=IL26%20%20AND%20Homo) | interleukin 26 | 1.66E-03 | -2.04 |
| 2445982 | NM_004673 | [ANGPTL1](http://www.ncbi.nlm.nih.gov/sites/entrez?db=gene&cmd=&term=ANGPTL1%20%20AND%20Homo) | angiopoietin-like 1 | 3.46E-03 | -2.00 |
| 2895244 | NM_001955 | [EDN1](http://www.ncbi.nlm.nih.gov/sites/entrez?db=gene&cmd=&term=EDN1%20%20AND%20Homo) | endothelin 1 | 2.27E-04 | 2.01 |
| 2815018 | NM_198486 | [RPL7L1](http://www.ncbi.nlm.nih.gov/sites/entrez?db=gene&cmd=&term=RPL7L1%20%20AND%20Homo) | ribosomal protein L7-like 1 | 3.30E-03 | 2.02 |
| 3756750 | NM_031854 | [KRTAP4-12](http://www.ncbi.nlm.nih.gov/sites/entrez?db=gene&cmd=&term=KRTAP4-12%20%20AND%20Homo) | keratin associated protein 4-12 | 6.06E-03 | 2.04 |
| 2429371 | NM_005725 | [TSPAN2](http://www.ncbi.nlm.nih.gov/sites/entrez?db=gene&cmd=&term=TSPAN2%20%20AND%20Homo) | tetraspanin 2 | 1.32E-03 | 2.05 |
| 2395177 | NM_018948 | [ERRFI1](http://www.ncbi.nlm.nih.gov/sites/entrez?db=gene&cmd=&term=ERRFI1%20%20AND%20Homo) | ERBB receptor feedback inhibitor 1 | 7.45E-05 | 2.06 |
| 3020222 | AY143171 | [LOC100128868](http://www.ncbi.nlm.nih.gov/sites/entrez?db=gene&cmd=&term=LOC100128868%20%20AND%20Homo) | testin-related protein TRG | 4.24E-04 | 2.06 |
| 2913444 | NM_019842 | [KCNQ5](http://www.ncbi.nlm.nih.gov/sites/entrez?db=gene&cmd=&term=KCNQ5%20%20AND%20Homo) | potassium voltage-gated channel, KQT-like subfamily, member 5 | 1.38E-03 | 2.07 |
| 3869215 | NM_001523 | [HAS1](http://www.ncbi.nlm.nih.gov/sites/entrez?db=gene&cmd=&term=HAS1%20%20AND%20Homo) | hyaluronan synthase 1 | 1.22E-03 | 2.08 |
| 3488592 | NM_005694 | [COX17](http://www.ncbi.nlm.nih.gov/sites/entrez?db=gene&cmd=&term=COX17%20%20AND%20Homo) | COX17 cytochrome c oxidase assembly homolog (S. cerevisiae) | 4.67E-04 | 2.10 |
| 3631397 | NM_018003 | [UACA](http://www.ncbi.nlm.nih.gov/sites/entrez?db=gene&cmd=&term=UACA%20%20AND%20Homo) | uveal autoantigen with coiled-coil domains and ankyrin repeats | 1.98E-04 | 2.10 |
| 2925953 | NM_006208 | [ENPP1](http://www.ncbi.nlm.nih.gov/sites/entrez?db=gene&cmd=&term=ENPP1%20%20AND%20Homo) | ectonucleotide pyrophosphatase/phosphodiesterase 1 | 8.13E-04 | 2.13 |
| 2926323 | NM_004100 | [EYA4](http://www.ncbi.nlm.nih.gov/sites/entrez?db=gene&cmd=&term=EYA4%20%20AND%20Homo) | eyes absent homolog 4 (Drosophila) | 1.67E-04 | 2.14 |
| 3436329 | NM_181709 | [FAM101A](http://www.ncbi.nlm.nih.gov/sites/entrez?db=gene&cmd=&term=FAM101A%20%20AND%20Homo) | family with sequence similarity 101, member A | 9.30E-05 | 2.14 |
| 3930525 | NR_026812 | [C21orf96](http://www.ncbi.nlm.nih.gov/sites/entrez?db=gene&cmd=&term=C21orf96%20%20AND%20Homo) | chromosome 21 open reading frame 96 | 2.47E-04 | 2.15 |
| 3266408 | NM_004098 | [EMX2](http://www.ncbi.nlm.nih.gov/sites/entrez?db=gene&cmd=&term=EMX2%20%20AND%20Homo) | empty spiracles homeobox 2 | 1.61E-07 | 2.16 |
| 2886679 | NM_004137 | [KCNMB1](http://www.ncbi.nlm.nih.gov/sites/entrez?db=gene&cmd=&term=KCNMB1%20%20AND%20Homo) | potassium large conductance calcium-activated channel, subfamily M, beta member 1 | 1.88E-04 | 2.17 |
| 3049522 | NM_022748 | [TNS3](http://www.ncbi.nlm.nih.gov/sites/entrez?db=gene&cmd=&term=TNS3%20%20AND%20Homo) | tensin 3 | 1.81E-05 | 2.17 |
| 2733360 | NM_004464 | [FGF5](http://www.ncbi.nlm.nih.gov/sites/entrez?db=gene&cmd=&term=FGF5%20%20AND%20Homo) | fibroblast growth factor 5 | 6.07E-05 | 2.18 |
| 2783207 | NM_003619 | [PRSS12](http://www.ncbi.nlm.nih.gov/sites/entrez?db=gene&cmd=&term=PRSS12%20%20AND%20Homo) | protease, serine, 12 (neurotrypsin, motopsin) | 2.21E-04 | 2.22 |
| 2632225 | NM_005233 | [EPHA3](http://www.ncbi.nlm.nih.gov/sites/entrez?db=gene&cmd=&term=EPHA3%20%20AND%20Homo) | EPH receptor A3 | 9.69E-04 | 2.22 |
| 2337740 | NM_006252 | [PRKAA2](http://www.ncbi.nlm.nih.gov/sites/entrez?db=gene&cmd=&term=PRKAA2%20%20AND%20Homo) | protein kinase, AMP-activated, alpha 2 catalytic subunit | 5.70E-04 | 2.23 |
| 3973768 | NM_198511 | [LANCL3](http://www.ncbi.nlm.nih.gov/sites/entrez?db=gene&cmd=&term=LANCL3%20%20AND%20Homo) | LanC lantibiotic synthetase component C-like 3 (bacterial) | 8.35E-05 | 2.26 |
| 3023883 | NM_016352 | [CPA4](http://www.ncbi.nlm.nih.gov/sites/entrez?db=gene&cmd=&term=CPA4%20%20AND%20Homo) | carboxypeptidase A4 | 6.52E-05 | 2.26 |
| 2856995 | NM_007036 | [ESM1](http://www.ncbi.nlm.nih.gov/sites/entrez?db=gene&cmd=&term=ESM1%20%20AND%20Homo) | endothelial cell-specific molecule 1 | 1.23E-03 | 2.27 |
| 2449104 | NM_003783 | [B3GALT2](http://www.ncbi.nlm.nih.gov/sites/entrez?db=gene&cmd=&term=B3GALT2%20%20AND%20Homo) | UDP-Gal:betaGlcNAc beta 1,3-galactosyltransferase, polypeptide 2 | 4.52E-05 | 2.27 |
| 3424785 | NM_006982 | [ALX1](http://www.ncbi.nlm.nih.gov/sites/entrez?db=gene&cmd=&term=ALX1%20%20AND%20Homo) | ALX homeobox 1 | 4.29E-06 | 2.28 |
| 2710474 | NM_018192 | [LEPREL1](http://www.ncbi.nlm.nih.gov/sites/entrez?db=gene&cmd=&term=LEPREL1%20%20AND%20Homo) | leprecan-like 1 | 6.17E-05 | 2.30 |
| 2913442 | NM_019842 | [KCNQ5](http://www.ncbi.nlm.nih.gov/sites/entrez?db=gene&cmd=&term=KCNQ5%20%20AND%20Homo) | potassium voltage-gated channel, KQT-like subfamily, member 5 | 6.83E-03 | 2.30 |
| 3875908 | NM_001172646 | [PLCB4](http://www.ncbi.nlm.nih.gov/sites/entrez?db=gene&cmd=&term=PLCB4%20%20AND%20Homo) | phospholipase C, beta 4 | 5.36E-04 | 2.35 |
| 3686503 | NM_001037808 | [EIF3C](http://www.ncbi.nlm.nih.gov/sites/entrez?db=gene&cmd=&term=EIF3C%20%20AND%20Homo) | eukaryotic translation initiation factor 3, subunit C | 3.77E-03 | 2.35 |
| 3402786 | NM_000616 | [CD4](http://www.ncbi.nlm.nih.gov/sites/entrez?db=gene&cmd=&term=CD4%20%20AND%20Homo) | CD4 molecule | 6.46E-06 | 2.36 |
| 2412624 | NM_002867 | [RAB3B](http://www.ncbi.nlm.nih.gov/sites/entrez?db=gene&cmd=&term=RAB3B%20%20AND%20Homo) | RAB3B, member RAS oncogene family | 3.36E-04 | 2.36 |
| 2835123 | NR_027180 | [LOC728264](http://www.ncbi.nlm.nih.gov/sites/entrez?db=gene&cmd=&term=LOC728264%20%20AND%20Homo) | hypothetical LOC728264 | 3.43E-03 | 2.37 |
| 2326237 | NM_004455 | [EXTL1](http://www.ncbi.nlm.nih.gov/sites/entrez?db=gene&cmd=&term=EXTL1%20%20AND%20Homo) | exostoses (multiple)-like 1 | 1.54E-04 | 2.39 |
| 2893794 | NM_004415 | [DSP](http://www.ncbi.nlm.nih.gov/sites/entrez?db=gene&cmd=&term=DSP%20%20AND%20Homo) | desmoplakin | 1.62E-05 | 2.44 |
| 2805078 | NM_004932 | [CDH6](http://www.ncbi.nlm.nih.gov/sites/entrez?db=gene&cmd=&term=CDH6%20%20AND%20Homo) | cadherin 6, type 2, K-cadherin (fetal kidney) | 3.62E-04 | 2.44 |
| 2879166 | NR_026695 | [FGF1](http://www.ncbi.nlm.nih.gov/sites/entrez?db=gene&cmd=&term=FGF1%20%20AND%20Homo) | fibroblast growth factor 1 (acidic) | 1.44E-04 | 2.47 |
| 3662130 | NR_001447 // NR_001447 | [MT1L // MT1L](http://www.ncbi.nlm.nih.gov/sites/entrez?db=gene&cmd=&term=MT1L%20%20//%20MT1L%20%20AND%20Homo) | metallothionein 1L (gene/pseudogene) // metallothionein 1L (gene/pseudogene) | 4.40E-03 | 2.49 |
| 3042919 | NM_152739 | [HOXA9](http://www.ncbi.nlm.nih.gov/sites/entrez?db=gene&cmd=&term=HOXA9%20%20AND%20Homo) | homeobox A9 | 4.09E-11 | 2.49 |
| 2527253 | NM_000597 | [IGFBP2](http://www.ncbi.nlm.nih.gov/sites/entrez?db=gene&cmd=&term=IGFBP2%20%20AND%20Homo) | insulin-like growth factor binding protein 2, 36kDa | 5.04E-03 | 2.50 |
| 3905145 | NM_004613 | [TGM2](http://www.ncbi.nlm.nih.gov/sites/entrez?db=gene&cmd=&term=TGM2%20%20AND%20Homo) | transglutaminase 2 (C polypeptide, protein-glutamine-gamma-glutamyltransferase) | 5.81E-04 | 2.53 |
| 3150455 | NM_002546 | [TNFRSF11B](http://www.ncbi.nlm.nih.gov/sites/entrez?db=gene&cmd=&term=TNFRSF11B%20%20AND%20Homo) | tumor necrosis factor receptor superfamily, member 11b | 2.04E-04 | 2.55 |
| 2835614 | ENST00000394243 | [SYNPO](http://www.ncbi.nlm.nih.gov/sites/entrez?db=gene&cmd=&term=SYNPO%20%20AND%20Homo) | synaptopodin | 6.96E-04 | 2.56 |
| 2390655 | AK303004 | [FLJ45445](http://www.ncbi.nlm.nih.gov/sites/entrez?db=gene&cmd=&term=FLJ45445%20%20AND%20Homo) | hypothetical LOC399844 | 7.97E-04 | 2.60 |
| 2835119 | NR_027180 | [LOC728264](http://www.ncbi.nlm.nih.gov/sites/entrez?db=gene&cmd=&term=LOC728264%20%20AND%20Homo) | hypothetical LOC728264 | 3.07E-03 | 2.62 |
| 2364381 | NM_001102445 | [RGS4](http://www.ncbi.nlm.nih.gov/sites/entrez?db=gene&cmd=&term=RGS4%20%20AND%20Homo) | regulator of G-protein signaling 4 | 4.28E-03 | 2.63 |
| 3279313 | NM_003638 | [ITGA8](http://www.ncbi.nlm.nih.gov/sites/entrez?db=gene&cmd=&term=ITGA8%20%20AND%20Homo) | integrin, alpha 8 | 2.58E-03 | 2.73 |
| 3631394 | NM_018003 | [UACA](http://www.ncbi.nlm.nih.gov/sites/entrez?db=gene&cmd=&term=UACA%20%20AND%20Homo) | uveal autoantigen with coiled-coil domains and ankyrin repeats | 3.30E-04 | 2.75 |
| 2348992 | NM_001078 | [VCAM1](http://www.ncbi.nlm.nih.gov/sites/entrez?db=gene&cmd=&term=VCAM1%20%20AND%20Homo) | vascular cell adhesion molecule 1 | 8.08E-03 | 2.83 |
| 3550307 | NM_000710 | [BDKRB1](http://www.ncbi.nlm.nih.gov/sites/entrez?db=gene&cmd=&term=BDKRB1%20%20AND%20Homo) | bradykinin receptor B1 | 3.95E-06 | 2.84 |
| 3128046 | NM_003155 | [STC1](http://www.ncbi.nlm.nih.gov/sites/entrez?db=gene&cmd=&term=STC1%20%20AND%20Homo) | stanniocalcin 1 | 8.91E-03 | 2.90 |
| 2835125 | NR_029686 | [MIR145](http://www.ncbi.nlm.nih.gov/sites/entrez?db=gene&cmd=&term=MIR145%20%20AND%20Homo) | microRNA 145 | 1.48E-03 | 2.91 |
| 3394264 | NM_006500 | [MCAM](http://www.ncbi.nlm.nih.gov/sites/entrez?db=gene&cmd=&term=MCAM%20%20AND%20Homo) | melanoma cell adhesion molecule | 5.14E-05 | 2.93 |
| 2853426 | NM_001161429 | [RANBP3L](http://www.ncbi.nlm.nih.gov/sites/entrez?db=gene&cmd=&term=RANBP3L%20%20AND%20Homo) | RAN binding protein 3-like | 9.52E-04 | 2.95 |
| 3726154 | NM_002204 | [ITGA3](http://www.ncbi.nlm.nih.gov/sites/entrez?db=gene&cmd=&term=ITGA3%20%20AND%20Homo) | integrin, alpha 3 (antigen CD49C, alpha 3 subunit of VLA-3 receptor) | 5.88E-06 | 2.97 |
| 3452478 | NM_001143668 | [AMIGO2](http://www.ncbi.nlm.nih.gov/sites/entrez?db=gene&cmd=&term=AMIGO2%20%20AND%20Homo) | adhesion molecule with Ig-like domain 2 | 6.30E-04 | 3.02 |
| 3756723 | NM_033184 | [KRTAP2-4](http://www.ncbi.nlm.nih.gov/sites/entrez?db=gene&cmd=&term=KRTAP2-4%20%20AND%20Homo) | keratin associated protein 2-4 | 8.24E-04 | 3.09 |
| 2590715 | NM_001463 | [FRZB](http://www.ncbi.nlm.nih.gov/sites/entrez?db=gene&cmd=&term=FRZB%20%20AND%20Homo) | frizzled-related protein | 1.34E-03 | 3.10 |
| 2731496 | NM_001013442 | [EPGN](http://www.ncbi.nlm.nih.gov/sites/entrez?db=gene&cmd=&term=EPGN%20%20AND%20Homo) | epithelial mitogen homolog (mouse) | 1.45E-03 | 3.12 |
| 3416290 | NM_017409 | [HOXC10](http://www.ncbi.nlm.nih.gov/sites/entrez?db=gene&cmd=&term=HOXC10%20%20AND%20Homo) | homeobox C10 | 1.78E-08 | 3.13 |
| 3262129 | NM_032727 | [INA](http://www.ncbi.nlm.nih.gov/sites/entrez?db=gene&cmd=&term=INA%20%20AND%20Homo) | internexin neuronal intermediate filament protein, alpha | 5.59E-04 | 3.15 |
| 3299970 | NM_014391 | [ANKRD1](http://www.ncbi.nlm.nih.gov/sites/entrez?db=gene&cmd=&term=ANKRD1%20%20AND%20Homo) | ankyrin repeat domain 1 (cardiac muscle) | 2.93E-04 | 3.15 |
| 2398820 | NM_007365 | [PADI2](http://www.ncbi.nlm.nih.gov/sites/entrez?db=gene&cmd=&term=PADI2%20%20AND%20Homo) | peptidyl arginine deiminase, type II | 5.66E-03 | 3.16 |
| 3063035 | NM_001134450 | [TMEM130](http://www.ncbi.nlm.nih.gov/sites/entrez?db=gene&cmd=&term=TMEM130%20%20AND%20Homo) | transmembrane protein 130 | 6.65E-05 | 3.24 |
| 4015312 | NM_001105243 | [PCDH19](http://www.ncbi.nlm.nih.gov/sites/entrez?db=gene&cmd=&term=PCDH19%20%20AND%20Homo) | protocadherin 19 | 2.52E-07 | 3.42 |
| 2970525 | BC037331 | [LOC285758](http://www.ncbi.nlm.nih.gov/sites/entrez?db=gene&cmd=&term=LOC285758%20%20AND%20Homo) | hypothetical protein LOC285758 | 1.60E-06 | 3.64 |
| 3756689 | NM_030967 | [KRTAP1-1](http://www.ncbi.nlm.nih.gov/sites/entrez?db=gene&cmd=&term=KRTAP1-1%20%20AND%20Homo) | keratin associated protein 1-1 | 3.30E-08 | 3.69 |
| 2509988 | NM_177964 | [LYPD6B](http://www.ncbi.nlm.nih.gov/sites/entrez?db=gene&cmd=&term=LYPD6B%20%20AND%20Homo) | LY6/PLAUR domain containing 6B | 8.15E-06 | 3.79 |
| 3277110 | NR_027082 | [SFTA1P](http://www.ncbi.nlm.nih.gov/sites/entrez?db=gene&cmd=&term=SFTA1P%20%20AND%20Homo) | surfactant associated 1 (pseudogene) | 1.18E-03 | 4.00 |
| 3756676 | NM_031957 // NM_031957 // NM_031957 | [KRTAP1-5 // KRTAP1-5 // KRTAP1-5](http://www.ncbi.nlm.nih.gov/sites/entrez?db=gene&cmd=&term=KRTAP1-5%20%20//%20KRTAP1-5%20%20//%20KRTAP1-5%20%20AND%20Homo) | keratin associated protein 1-5 // keratin associated protein 1-5 // keratin associated protein 1-5 | 2.48E-05 | 4.14 |
| 2743800 | NM_032961 | [PCDH10](http://www.ncbi.nlm.nih.gov/sites/entrez?db=gene&cmd=&term=PCDH10%20%20AND%20Homo) | protocadherin 10 | 9.20E-04 | 4.27 |
| 2990404 | NM_001112706 | [SCIN](http://www.ncbi.nlm.nih.gov/sites/entrez?db=gene&cmd=&term=SCIN%20%20AND%20Homo) | scinderin | 2.12E-05 | 4.32 |
| 2602304 | NM_024795 | [TM4SF20](http://www.ncbi.nlm.nih.gov/sites/entrez?db=gene&cmd=&term=TM4SF20%20%20AND%20Homo) | transmembrane 4 L six family member 20 | 2.10E-03 | 4.41 |
| 3786868 | NM_001128588 | [SLC14A1](http://www.ncbi.nlm.nih.gov/sites/entrez?db=gene&cmd=&term=SLC14A1%20%20AND%20Homo) | solute carrier family 14 (urea transporter), member 1 (Kidd blood group) | 4.16E-06 | 5.28 |
| 2435649 | NM_002016 | [FLG](http://www.ncbi.nlm.nih.gov/sites/entrez?db=gene&cmd=&term=FLG%20%20AND%20Homo) | filaggrin | 2.52E-06 | 7.50 |
