# Supplementary material for: Citrullination of histone H3 drives IL-6 production by bone marrow mesenchymal stem cells in MGUS and multiple myeloma
Source: Leukemia. 2016 Aug 12;31(2):373–81. doi: 10.1038/leu.2016.187 (PMC5292682; doi:10.1038/leu.2016.187)
Supplement: Supplementary Table 7 [file leu2016187x7.docx]

| **Probe Set ID** | **Gene Accession** | **Gene Symbol** | **Gene Description** | **P Value** | **Fold Change** |
| --- | --- | --- | --- | --- | --- |
| 2704998 | Z19588 | [SKIL](http://www.ncbi.nlm.nih.gov/sites/entrez?db=gene&cmd=&term=SKIL%20%20AND%20Homo) | SKI-like oncogene | 2.66E-03 | -1.68 |
| 2848257 | AK124354 | [CCT5](http://www.ncbi.nlm.nih.gov/sites/entrez?db=gene&cmd=&term=CCT5%20%20AND%20Homo) | chaperonin containing TCP1, subunit 5 (epsilon) | 1.18E-03 | -1.60 |
| 3982023 | NM_016120 | [RLIM](http://www.ncbi.nlm.nih.gov/sites/entrez?db=gene&cmd=&term=RLIM%20%20AND%20Homo) | ring finger protein, LIM domain interacting | 9.60E-03 | -1.58 |
| 2570158 | NM_018181 | [ZNF532](http://www.ncbi.nlm.nih.gov/sites/entrez?db=gene&cmd=&term=ZNF532%20%20AND%20Homo) | zinc finger protein 532 | 5.56E-03 | -1.54 |
| 2528123 | AK093857 | [LOC285181](http://www.ncbi.nlm.nih.gov/sites/entrez?db=gene&cmd=&term=LOC285181%20%20AND%20Homo) | hypothetical protein LOC285181 | 1.54E-03 | -1.54 |
| 4036348 | NM_001006121 | [RBMY1B](http://www.ncbi.nlm.nih.gov/sites/entrez?db=gene&cmd=&term=RBMY1B%20%20AND%20Homo) | RNA binding motif protein, Y-linked, family 1, member B | 2.90E-03 | -1.51 |
| 3650511 | NM_000971 | [RPL7](http://www.ncbi.nlm.nih.gov/sites/entrez?db=gene&cmd=&term=RPL7%20%20AND%20Homo) | ribosomal protein L7 | 3.22E-03 | -1.50 |
| 2361564 | NM_001105669 | [TTC24](http://www.ncbi.nlm.nih.gov/sites/entrez?db=gene&cmd=&term=TTC24%20%20AND%20Homo) | tetratricopeptide repeat domain 24 | 8.44E-03 | -1.49 |
| 2938955 | XM_002345871 | [LOC100288968](http://www.ncbi.nlm.nih.gov/sites/entrez?db=gene&cmd=&term=LOC100288968%20%20AND%20Homo) | hypothetical protein LOC100288968 | 1.38E-03 | -1.46 |
| 3793825 | NM_018235 | [CNDP2](http://www.ncbi.nlm.nih.gov/sites/entrez?db=gene&cmd=&term=CNDP2%20%20AND%20Homo) | CNDP dipeptidase 2 (metallopeptidase M20 family) | 1.95E-03 | -1.41 |
| 2367015 | NM_001460 | [FMO2](http://www.ncbi.nlm.nih.gov/sites/entrez?db=gene&cmd=&term=FMO2%20%20AND%20Homo) | flavin containing monooxygenase 2 (non-functional) | 1.04E-03 | -1.37 |
| 3393306 | XM_001725443 | [LOC100129201](http://www.ncbi.nlm.nih.gov/sites/entrez?db=gene&cmd=&term=LOC100129201%20%20AND%20Homo) | hypothetical protein LOC100129201 | 9.78E-03 | -1.36 |
| 3086312 | AK091259 | [LOC649294](http://www.ncbi.nlm.nih.gov/sites/entrez?db=gene&cmd=&term=LOC649294%20%20AND%20Homo) | hypothetical LOC649294 | 8.58E-03 | -1.33 |
| 2718452 | XM_002346604 | [LOC100287951](http://www.ncbi.nlm.nih.gov/sites/entrez?db=gene&cmd=&term=LOC100287951%20%20AND%20Homo) | dynein, light chain, Tctex-type 1 pseudogene | 1.90E-03 | -1.30 |
| 3177809 | NM_001255 | [CDC20](http://www.ncbi.nlm.nih.gov/sites/entrez?db=gene&cmd=&term=CDC20%20%20AND%20Homo) | cell division cycle 20 homolog (S. cerevisiae) | 6.83E-04 | 1.30 |
| 2856484 | NM_176806 | [MOCS2](http://www.ncbi.nlm.nih.gov/sites/entrez?db=gene&cmd=&term=MOCS2%20%20AND%20Homo) | molybdenum cofactor synthesis 2 | 3.06E-03 | 1.30 |
| 3050823 | NR_026999 | [LOC349114](http://www.ncbi.nlm.nih.gov/sites/entrez?db=gene&cmd=&term=LOC349114%20%20AND%20Homo) | hypothetical LOC349114 | 6.09E-03 | 1.31 |
| 2531589 | NM_030926 | [ITM2C](http://www.ncbi.nlm.nih.gov/sites/entrez?db=gene&cmd=&term=ITM2C%20%20AND%20Homo) | integral membrane protein 2C | 3.27E-04 | 1.31 |
| 3673892 | NM_004933 | [CDH15](http://www.ncbi.nlm.nih.gov/sites/entrez?db=gene&cmd=&term=CDH15%20%20AND%20Homo) | cadherin 15, type 1, M-cadherin (myotubule) | 1.01E-03 | 1.33 |
| 3467235 | AK092631 | [LOC121456](http://www.ncbi.nlm.nih.gov/sites/entrez?db=gene&cmd=&term=LOC121456%20%20AND%20Homo) | similar to solute carrier family 9, member 7 | 5.49E-03 | 1.34 |
| 3940580 | BC047380 | [CRYBB2P1](http://www.ncbi.nlm.nih.gov/sites/entrez?db=gene&cmd=&term=CRYBB2P1%20%20AND%20Homo) | crystallin, beta B2 pseudogene 1 | 8.34E-03 | 1.34 |
| 3949009 | AK091355 | [LOC284926](http://www.ncbi.nlm.nih.gov/sites/entrez?db=gene&cmd=&term=LOC284926%20%20AND%20Homo) | hypothetical protein LOC284926 | 8.72E-03 | 1.36 |
| 2444060 | NR_029618 | [MIR199A2](http://www.ncbi.nlm.nih.gov/sites/entrez?db=gene&cmd=&term=MIR199A2%20%20AND%20Homo) | microRNA 199a-2 | 5.44E-03 | 1.37 |
| 2967687 | NM_024102 | [WDR77](http://www.ncbi.nlm.nih.gov/sites/entrez?db=gene&cmd=&term=WDR77%20%20AND%20Homo) | WD repeat domain 77 | 2.64E-03 | 1.38 |
| 3856036 | ENST00000443905 | [ZNF506](http://www.ncbi.nlm.nih.gov/sites/entrez?db=gene&cmd=&term=ZNF506%20%20AND%20Homo) | zinc finger protein 506 | 2.09E-03 | 1.40 |
| 3805952 | XM_001716741 | [LOC100131669](http://www.ncbi.nlm.nih.gov/sites/entrez?db=gene&cmd=&term=LOC100131669%20%20AND%20Homo) | hypothetical LOC100131669 | 7.72E-04 | 1.41 |
| 2817212 | NM_017614 | [BHMT2](http://www.ncbi.nlm.nih.gov/sites/entrez?db=gene&cmd=&term=BHMT2%20%20AND%20Homo) | betaine--homocysteine S-methyltransferase 2 | 1.63E-03 | 1.47 |
| 2735129 | NM_004967 | [IBSP](http://www.ncbi.nlm.nih.gov/sites/entrez?db=gene&cmd=&term=IBSP%20%20AND%20Homo) | integrin-binding sialoprotein | 8.59E-03 | 1.49 |
| 2701242 | NR_027038 | [LOC401093](http://www.ncbi.nlm.nih.gov/sites/entrez?db=gene&cmd=&term=LOC401093%20%20AND%20Homo) | hypothetical LOC401093 | 3.98E-04 | 1.49 |
| 3888522 | NM_005985 | [SNAI1](http://www.ncbi.nlm.nih.gov/sites/entrez?db=gene&cmd=&term=SNAI1%20%20AND%20Homo) | snail homolog 1 (Drosophila) | 4.44E-03 | 1.59 |
| 2758870 | NM_018659 | [CYTL1](http://www.ncbi.nlm.nih.gov/sites/entrez?db=gene&cmd=&term=CYTL1%20%20AND%20Homo) | cytokine-like 1 | 1.66E-03 | 1.95 |
