# Supplementary material for: Citrullination of histone H3 drives IL-6 production by bone marrow mesenchymal stem cells in MGUS and multiple myeloma
Source: Leukemia. 2016 Aug 12;31(2):373–81. doi: 10.1038/leu.2016.187 (PMC5292682; doi:10.1038/leu.2016.187)
Supplement: Supplementary Table 8 [file leu2016187x8.docx]

Supplementary Table 8. Pathways over-represented in genes differentially expressed between myeloma and control p<0.05.

| **Pathway** | **P-value** | **Differentially expressed genes** |
| --- | --- | --- |
| Wnt signaling pathway | 0.00012 | CDH6, CSNK1D, EDN1, FAT3, HOXA5, HOXC6, PCDH10, PCDH19, PLCB4, SFRP2, SFRP4 |
| Blood coagulation | 0.01150 | F2R, F2RL2, THBD |
| Cell cycle | 0.02020 | EIF3F, PSME2 |
| Angiogenesis | 0.02680 | EPHA3, FGF1, PDGFA, PDGFD, RASA1 |
| Hedgehog signaling pathway | 0.02910 | CSNK1D, GLI3 |
| Cadherin signaling pathway | 0.03120 | CDH6,FAT3,FAT3,PCDH10,PCDH19 |
