# Supplementary material for: Citrullination of histone H3 drives IL-6 production by bone marrow mesenchymal stem cells in MGUS and multiple myeloma
Source: Leukemia. 2016 Aug 12;31(2):373–81. doi: 10.1038/leu.2016.187 (PMC5292682; doi:10.1038/leu.2016.187)
Supplement: Supplementary Table 9 [file leu2016187x9.docx]

Supplementary Table 9. Pathways over-represented in genes differentially expressed between MGUS and control p<0.05.

| **Pathway** | **P-value** | **Differentially expressed genes** |
| --- | --- | --- |
| Wnt signaling pathway | 0.00002 | CDH6, CSNK1D, FAT3, HOXA5, HOXB5, HOXB6, HOXC6, PCDH10, SFRP2, SFRP4 |
| Hedgehog signaling pathway | 0.01320 | CSNK1D, GLI3 |
| Blood coagulation | 0.03710 | F2R, THBD |
| Cadherin signaling pathway | 0.04260 | CDH6, FAT3, PCDH10 |
