# Supplementary figures and images for: Citrullination of histone H3 drives IL-6 production by bone marrow mesenchymal stem cells in MGUS and multiple myeloma
Source: Leukemia. 2016 Aug 12;31(2):373–81. doi: 10.1038/leu.2016.187 (PMC5292682; doi:10.1038/leu.2016.187)

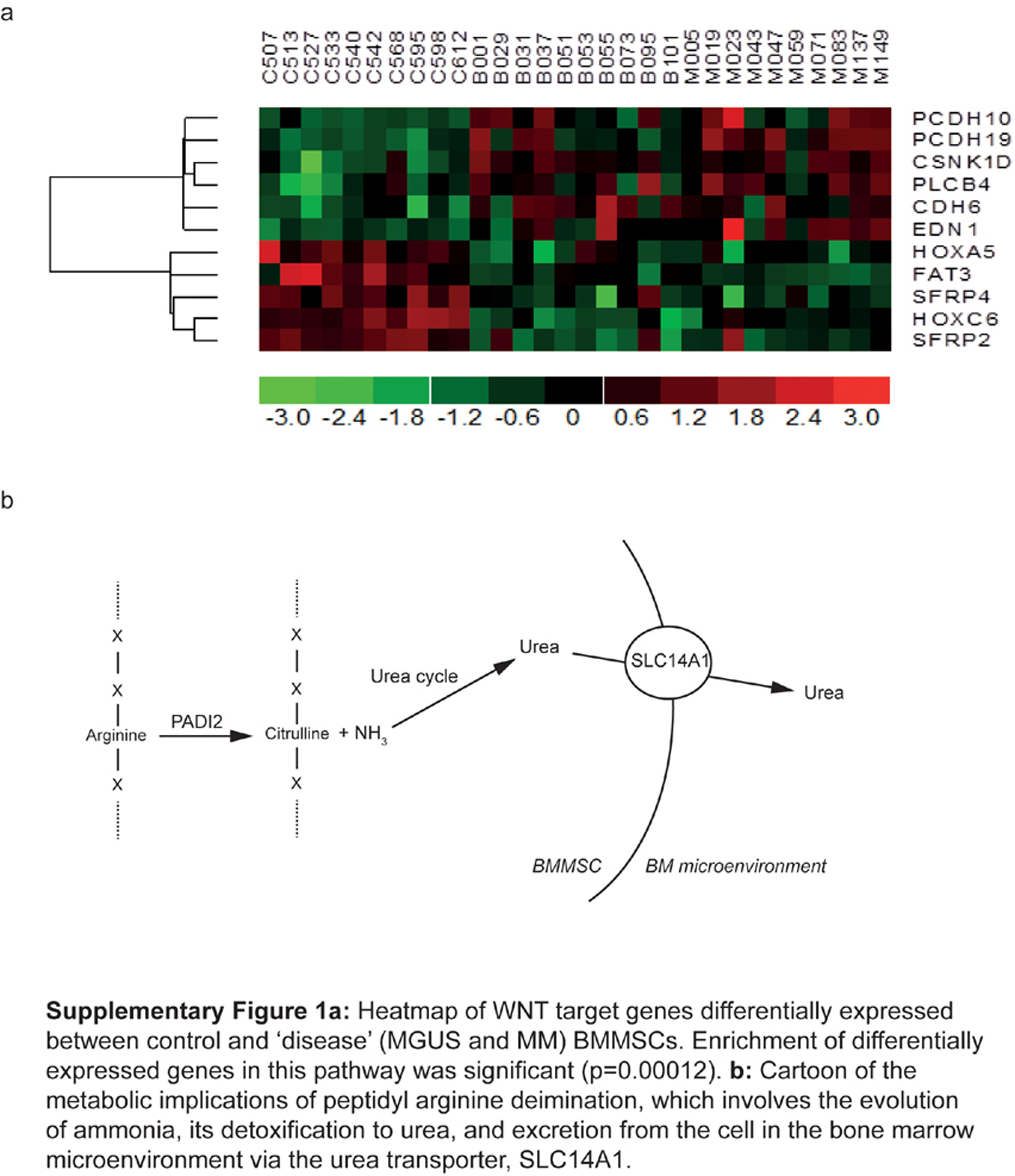

Supplement: Supplementary Figure 1 [file leu2016187x10.tif]

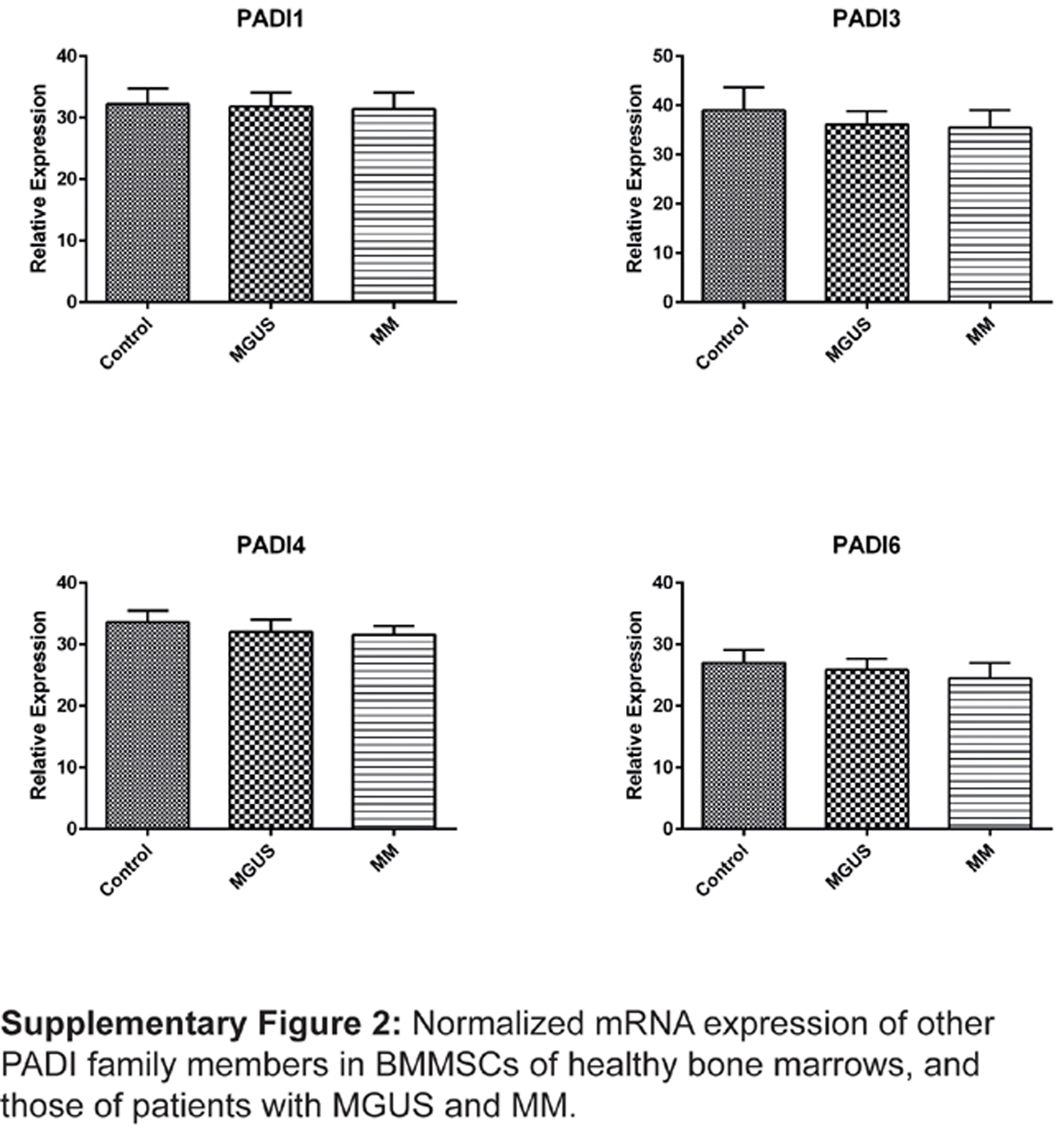

Supplement: Supplementary Figure 2 [file leu2016187x11.tif]

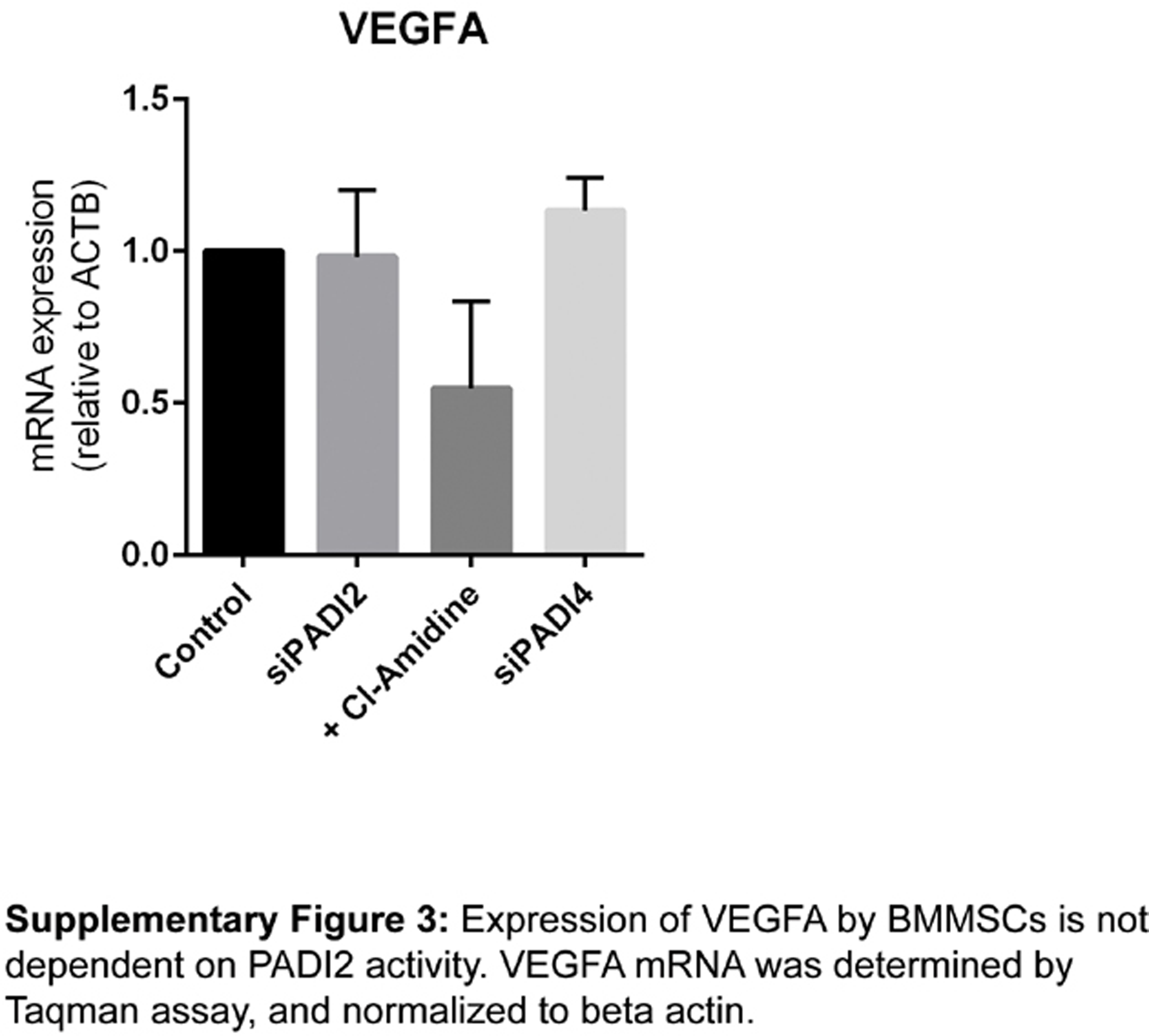

Supplement: Supplementary Figure 3 [file leu2016187x12.tif]
